# Gradual polyploid genome evolution revealed by pan-genomic analysis of *Brachypodium hybridum* and its diploid progenitors

**DOI:** 10.1038/s41467-020-17302-5

**Published:** 2020-07-29

**Authors:** Sean P. Gordon, Bruno Contreras-Moreira, Joshua J. Levy, Armin Djamei, Angelika Czedik-Eysenberg, Virginia S. Tartaglio, Adam Session, Joel Martin, Amy Cartwright, Andrew Katz, Vasanth R. Singan, Eugene Goltsman, Kerrie Barry, Vinh Ha Dinh-Thi, Boulos Chalhoub, Antonio Diaz-Perez, Ruben Sancho, Joanna Lusinska, Elzbieta Wolny, Candida Nibau, John H. Doonan, Luis A. J. Mur, Chris Plott, Jerry Jenkins, Samuel P. Hazen, Scott J. Lee, Shengqiang Shu, David Goodstein, Daniel Rokhsar, Jeremy Schmutz, Robert Hasterok, Pilar Catalan, John P. Vogel

**Affiliations:** 1 DOE Joint Genome Institute, Berkeley, CA 94720 USA; 2 Estación Experimental de Aula Dei (EEAD-CSIC), Zaragoza, Spain; 3 Fundación ARAID, Zaragoza, Spain; 4 Grupo de Bioquímica, Biofísica y Biología Computacional (BIFI, UNIZAR), Unidad Asociada al CSIC, Zaragoza, Spain; 5 University California, Berkeley, Berkeley, CA 94720 USA; 6 Gregor Mendel Institute of Molecular Plant Biology GmbH, Vienna, Austria; 7 Leibniz Institute of Plant Genetics and Crop Plant Research (IPK) Gatersleben. Stadt Seeland, Seeland, Germany; 8 Organization and evolution of complex genomes (OECG) Institut national de la Recherche agronomique (INRA), Université d’Evry Val d’Essonne (UEVE), Evry, France; 9 Institute of Crop Science, Zhejiang University, 866 Yu-Hang-Tang Road, 310058 Hangzhou, China; 10 Universidad de Zaragoza-Escuela Politécnica Superior de Huesca, 22071 Huesca, Spain; 11 Instituto de Genética, Facultad de Agronomía, Universidad Central de Venezuela, 2102 Maracay, Venezuela; 12 Plant Cytogenetics and Molecular Biology Group, Institute of Biology, Biotechnology and Environmental Protection, Faculty of Natural Sciences, University of Silesia in Katowice, 40-032 Katowice, Poland; 13 Institute of Biological, Environmental and Rural Sciences (IBERS), Aberystwyth University, Aberystwyth, Wales UK; 14 HudsonAlpha Institute for Biotechnology, Huntsville, AL 35806 USA; 15 Biology Department, University of Massachusetts Amherst, Amherst, MA 01003 USA; 16 Institute of Biology, Tomsk State University, Tomsk, 634050 Russia

**Keywords:** Comparative genomics, Polyploidy in plants, Natural variation in plants

## Abstract

Our understanding of polyploid genome evolution is constrained because we cannot know the exact founders of a particular polyploid. To differentiate between founder effects and post polyploidization evolution, we use a pan-genomic approach to study the allotetraploid *Brachypodium hybridum* and its diploid progenitors. Comparative analysis suggests that most *B. hybridum* whole gene presence/absence variation is part of the standing variation in its diploid progenitors. Analysis of nuclear single nucleotide variants, plastomes and k-mers associated with retrotransposons reveals two independent origins for *B. hybridum*, ~1.4 and ~0.14 million years ago. Examination of gene expression in the younger *B. hybridum* lineage reveals no bias in overall subgenome expression. Our results are consistent with a gradual accumulation of genomic changes after polyploidization and a lack of subgenome expression dominance. Significantly, if we did not use a pan-genomic approach, we would grossly overestimate the number of genomic changes attributable to post polyploidization evolution.

## Abstract (web summary)

Existing plant pan-genomic studies usually report considerable intraspecific whole gene presence-absence variation. Here, the authors use pan-genomic approach to reveal gradual polyploid genome evolution by analyzing of *Brachypodium hybridum* and its diploid progenitors.

## Introduction

Polyploidy is a major evolutionary force-shaping eukaryotic genomes, particularly in the flowering plants^1^. Indeed, the origin of all flowering plants can be traced back to a polyploidization event shortly before the emergence of flowers^2^. Thus, all flowering plants contain at least one polyploidization event in their evolutionary history and some, including the model plant *Arabidopsis thaliana*, contain several^3,4^. Over evolutionary time, polyploid genomes gradually revert to a functionally diploid state, by processes that include the shedding of redundant sequences. This process is still underway today, and 30–70% of all flowering plants are relatively recent polyploids whose genomes still contain multiple copies of many genes^5^. Included among these more recent polyploids are the species that feed the world (e.g., wheat, potato, sugarcane) and species that are increasingly being called upon as sources of renewable fuel (e.g., switchgrass). The pervasiveness of polyploidy indicates that it must be evolutionarily favorable, presumably because the sudden combination of genomes and the presence of duplicates for virtually every gene allows neofunctionalization and subfunctionalization as well as other benefits such as fixed heterosis^6^. In addition to its evolutionary role, polyploidy has been widely exploited in agriculture, and breeders have used artificially induced polyploidy to increase fruit and flower size (e.g., strawberry), create more adaptable and drought resistant crops (e.g., triticale) and produce seedless fruit (e.g., watermelon).

Despite the importance of polyploidy, detailed understanding of the temporal dynamics of genome evolution after polyploidization is still unknown. The current paradigm includes possible rapid epigenetic reprogramming, changes in gene expression, transposon activation, and altered gene splicing. Over longer timescales, changes in DNA sequence can lead to neofunctionalization, gene conversion, deletion or pseudogenization of nonessential genes^7,8^. In addition, dosage balance for some genes is expected to shape the genome at every stage via several mechanisms^9^. However, the precise order, timescale and the underlying mechanisms of these events are largely unknown^10^.

**Fig. 1:**
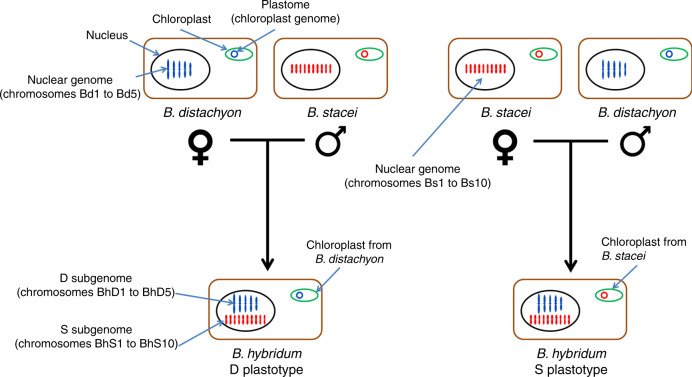
*Brachypodium hybridum* subgenome and plastotype naming convention. *Brachypodium hybridum* has formed more than once from reciprocal crosses. We can use chloroplast genome (plastome) sequence to determine the maternal progenitor species of any *B. hybridum* line. To facilitate writing about the origin of individual lines, we designate lines that had *B. distachyon* as a maternal progenitor species as D plastotype, and lines that had *B. stacei* as a maternal progenitor species as S plastotype. The chromosomes of *B. distachyon* are named Bd1 to Bd5, and the chromosomes of *B. stacei* are named Bs1 to Bs10. To allow us to easily link *B. hybridum* chromosomes to their diploid orthologs, we named *B. hybridum* chromosomes BhD1 to BhD5 for chromosomes derived from *B. distachyon* and BhS1 to BhS10 for chromosomes derived from *B. stacei*. The diagram shows stylized plant cells with color coding, indicating the origin of the plastome and nuclear genomes (*B. distachyon*-type, blue; *B. stacei*-type, red). For clarity, the haploid number of chromosomes is shown.

Further complicating our understanding of polyploid genome evolution is the high level of intraspecific genomic variation that has been detected by recent pan-genomic studies. Indeed, pan-genomes for several plant species have been shown to be much larger than the genomes of any individual line^11–15^. Thus, the standing variation in a polyploid species is the sum of the variation present in the parental genomes and the variation that developed after polyploidization^16^. This has important implications for our interpretation of polyploid genome evolution. If a polyploid species is formed from a single-hybridization event, then all the genomic diversity within that species will be due to sequence changes after the hybridization event. However, if the polyploid species was formed by multiple hybridizations, then the diversity would be the sum of the diversity in all the parents and the sequence changes that arose after the hybridization events^17^. In both scenarios, comparison of a single polyploid genome to single genomes of the diploid progenitor species will overestimate the variation due to evolution after the polyploidization event because of intraspecific differences between the reference diploid lines and the actual diploid parents of the polyploid. To overcome this limitation, it is necessary to use a pan-genomic approach. Unfortunately, polyploid biomass and grain crops are difficult experimental subjects because of their large, complex genomes; large physical size; and, for biomass crops, their typically outbreeding nature^18^. Therefore, we use a model grass polyploid system consisting of the diploids *Brachypodium distachyon* and *B. stacei*, and their derived allotetraploid *B. hybridum*. *B. distachyon* has emerged as a powerful model to study grass biology, and numerous experimental resources have been developed for it^19,20^. Historically, *B. distachyon* included the current *B. distachyon*, as well as *B. stacei* and *B. hybridum*. This complex was reclassified as three separate species^21–23^. Experimental crosses demonstrated that the three species are reproductively isolated from each other^22,24^. *B. hybridum* has subgenomes designated D and S that are derived from *B. distachyon* and *B. stacei*, respectively^25^. Because some *B. hybridum* lines have chloroplast genomes (plastomes) derived from *B. stacei* and some have plastomes derived from *B. distachyon*, we know that *B. hybridum* formed at least twice from reciprocal crosses^22,26^. *Brachypodium hybridum* lines are designated as D plastotype if their plastome is derived from *B. distachyon* or S plastotype if their plastome is derived from *B. stacei* (Fig. 1). All three species have very compact genomes (272, 234, and 509 Mb, respectively), small stature, and are genetically transformable and easily grown and manipulated in the laboratory^19^. In addition, as wild species their genomes have not been impacted by selection and genetic bottlenecks during domestication.

In this study, we compare multiple genomes to unveil two allopolyploidization events separated by over a million years that led to the same speciation outcome. We compare the D subgenomes of *B. hybridum* lines to the *B. distachyon* pan-genome to reveal that the vast majority of whole-gene presence/absence variation in *B. hybridum* is part of the standing variation of *B. distachyon*, indicating that even after 1.4 million years the process of post-polyploidization gene loss is still in its infancy. In contrast, we detect large numbers of small polymorphisms, SNVs (here we define SNV as a single-nucleotide variant, either within or between species, at a syntenically conserved position) and small insertions/deletions unique to each *B. hybridum* lineage, with much greater numbers present in the older lineage. This paints a picture of steady accumulation of small sequence changes, and very gradual gene loss during the first 1.4 million years after polyploidization in *B. hybridum*. Notably, if we compared a single polyploid genome to individual genomes of the diploid progenitors, we would reach very different conclusions.

## Results

### Nuclear genome assembly and annotation

In this study, we created chromosome-scale reference genomes for *B. stacei* and *B. hybridum*. We also created seven additional *B. hybridum* genome assemblies, including a high-quality PacBio-based assembly, and Illumina assemblies for six additional *B. hybridum* lines (Supplementary Data 1). In addition, we constructed Illumina-based assemblies for 57 *B. distachyon* lines (Supplementary Data 1). The *B. stacei* ABR114 genome was assembled from Illumina sequences using the ALLPATHS assembler. Contigs were ordered and oriented using a 22,043 marker genetic map to create ten chromosomes (named Bs1–Bs10) containing 234 Mb of sequence (Supplementary Data 2). The *B. hybridum* ABR113 reference genome was assembled similar to *B. stacei*, except the Meraculous assembler was used and additional improvement was done with Dovetail Genomic’s Chicago^TM^ library and HiRise^TM^ assembler followed by final orientation using a 45,197 marker genetic map (Supplementary Data 3). The chromosomes were named BhDN or BhSN where D or S refers to the *B. distachyon-*like or *B. stacei*-like subgenomes, respectively, and N stands for progenitor chromosome number (Fig. 1). The final assembly contains 99.4% (500.8/503.8 Mb) of all assembled sequence. The *B. hybridum* Bd28 genome was assembled from ~60× depth PacBio sequence using the Falcon assembler (Supplementary Data 1). The 765 contigs were ordered and oriented into 15 chromosomes based on synteny to *B. distachyon* (Bd21 v.3.1), *B. stacei* (ABR114 v.1.1), and *Sorghum bicolor* (Supplementary Data 1). The euchromatic portion of the genome assemblies was shown to be nearly complete by aligning RNA-seq (99% read alignment) and by BUSCO scores (~99%).

In addition to the high-quality genome assemblies, we created lower-quality assemblies for six *B. hybridum* lines using >60 × 250 bp Illumina paired-end reads (Supplementary Data 1). Genome assembly was performed with the Meraculous assembler. The initial scaffolds were binned into D and S subgenomes via the seal tool in bbtools (v37) using *B. distachyon* (v. 3.1) and *B. stacei* (v. 1.1). Similarly, we assembled genomes for 57 *B. distachyon* inbred lines from 250 bp paired-end Illumina reads (>50× coverage) from fragment insert libraries using the massively parallel HipMer genome assembler (Supplementary Data 1). Including 42 previously sequenced *B. distachyon* lines^15^, a total of 99 *B. distachyon* lines with fully assembled genomes were used to produce whole-genome multiple sequence alignments (Supplementary Data 4).

Assemblies from *B. hybridum* lines ABR113, Bd28, Bhyb30, Bhyb26, and Bhyb118-5; *B. distachyon* lines CSR-6 and LPA3.2; and *B. stacei* line ABR114 were annotated using the JGI annotation pipeline. Annotation metrics are listed in Supplementary Data 1.

### Synteny and karyotype analysis

**Fig. 2:**
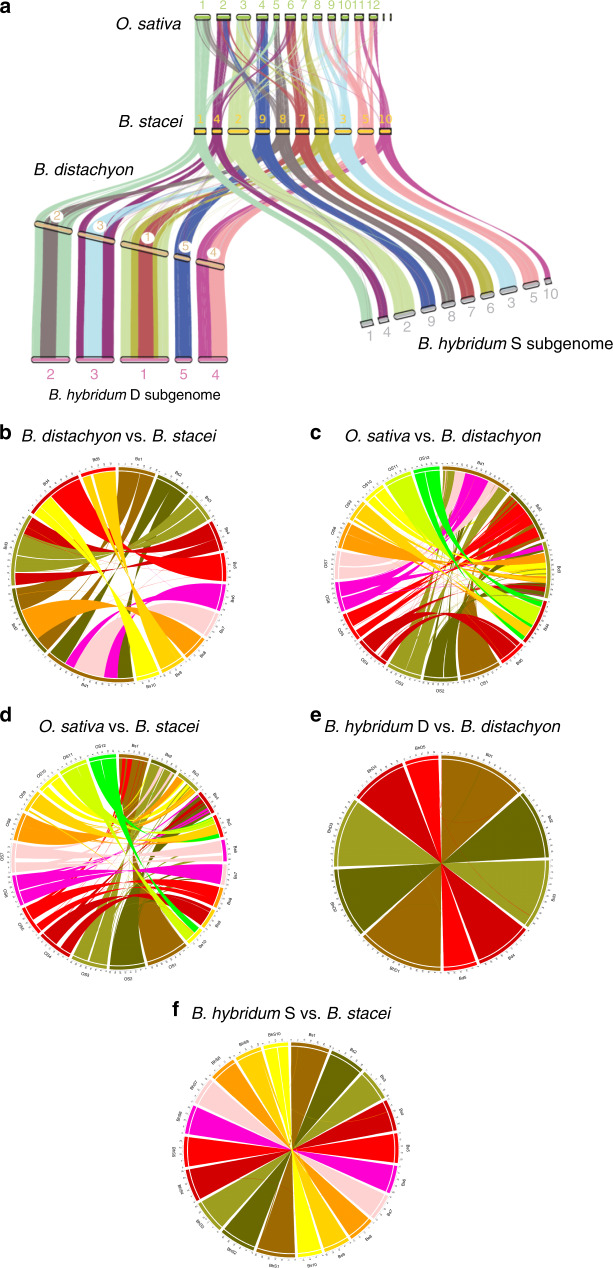
Syntenic relationships. **a** Synteny between chromosomes, represented by colors, indicates that the *B. distachyon* Bd21 chromosomes were derived from an ancestor similar to *B. stacei* ABR114 through a series of nested insertions of whole chromosomes into centromeric regions in Bd1, Bd2, and Bd3, fusion of two chromosomes to Bd4, and full collinearity of one chromosome with Bd5. Note that the genomes of *B. distachyon* Bd21 and *B. stacei* ABR114 are essentially 100% collinear with the S and D subgenomes of *B. hybridum* ABR113, respectively. **b**–**f** Circos plots showing syntenic relationships between the above-mentioned genomes and subgenomes. Note the high conservation in synteny between *B. distachyon* Bd21 and *B. hybridum* ABR113 D subgenome (**e**) and between *B. stacei* ABR114 and *B. hybridum* ABR113 S subgenome (**f**).

**Fig. 3:**
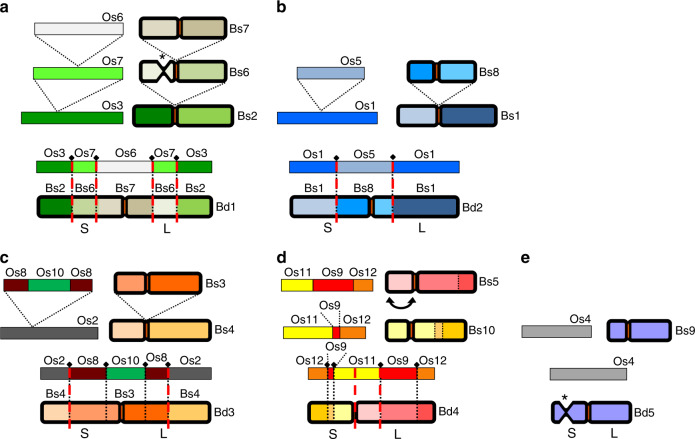
Chromosome evolution inferred from cytomolecular analysis. Color-coded representation of the syntenic relationship between the five *B. distachyon* Bd21 chromosomes and the corresponding *B. stacei* ABR114 chromosomes and ancestral rice chromosome equivalents. **a**–**c** The relationship between *B. distachyon* chromosomes Bd1 to Bd3 and the corresponding *B. stacei* chromosomes can be explained by a series of nested insertions of whole ancestral chromosome equivalents into centromeric regions (Bd1: Bs7 → Bs6 → Bs2; Bd2: Bs8 → Bs1; Bd3: Bs3 → Bs4). **d** Chromosome Bd4 shows the synteny of chromosomes Bs5 and Bs10 with the occurrence of a pericentromeric inversion in Bd4. **e** Chromosome Bd5 is highly collinear with Bs9. The asterisks indicate the location of the secondary constriction/satellite region in Bd5 and Bs6, which carry 35S rDNA loci.

We compared the genomes of *Oryza sativa* v7 (Phytozome, proteome id: 323), *B. stacei* ABR114, *B. distachyon* Bd21 v3.1, and *B. hybridum* ABR113 to examine chromosome structure and identify large-scale rearrangements in the *B. hybridum* subgenomes. Figure 2a, b shows that nested insertions of whole chromosomes, like the ones in *B. stacei*, into centromeric regions formed *B. distachyon* chromosomes Bd1–Bd3, as was noted previously^27^. In addition, Bd4 is syntenic with two *B. stacei* chromosomes, Bs5 and Bs10 (Fig. 2a, b), and three rice chromosomes, Os9, Os11, and Os12 (Figs. 2c and 3)^27^. Synteny suggests that Bs5 and Bs10 resulted either from the fission of a larger chromosome similar to Bd4 or by a reciprocal translocation followed by fusion of two ancestral chromosomes (Figs. 2b and 3). By contrast, Bd5 is highly collinear with chromosome Bs9, which in turn derives from large and small portions of Os4 and Os2, respectively (Fig. 2a–d). Comparison of *B. stacei* and *B. distachyon* chromosomes to the corresponding BhS and BhD chromosomes in *B. hybridum* ABR113 revealed almost perfect syntenic conservation (Fig. 2e, f). This indicates that there were no major rearrangements in the subgenomes after polyploidization.

We conducted molecular cytogenetic analysis to link the *B. stacei* and *B. hybridum* pseudomolecules to recently published karyotypes and validate the synteny analysis. Comparative chromosome barcoding using 80 low-repeat *B. distachyon* Bd21 BAC clones confirmed the synteny analysis (Fig. 3; Supplementary Fig. 1; Supplementary Table 1). The *B. stacei* chromosome numbers of the pseudomolecules differ from the numbers assigned by a recent karyotype because the pseudomolecules were numbered according to assembled length and the karyotype designations were based on optical characteristics (Supplementary Table 2)^28^. In this paper, we use the *B. stacei* chromosome numbers assigned to the pseudomolecules.

### Plastome sequencing

We assembled and annotated the plastomes of eight *B. hybridum* lines, 99 *B. distachyon* lines, and one *B. stacei* line (Supplementary Data 4). Two of the eight *B. hybridum* lines (Bhyb26 and Bhyb118-5) had *B. distachyon*-type plastomes (D plastotype), whereas the remaining *B. hybridum* lines had *B. stacei*-type plastomes (S plastotype) (Fig. 1). Plastome lengths varied from 135,031–135,423 bp in *B. distachyon* and the *B. hybridum* D-plastotype lines, and from 136,325–136,329 bp in *B. stacei* and the *B. hybridum-*S-plastotype lines (Supplementary Fig. 2). All *B. hybridum-*S-plastotype plastomes and *B. stacei* showed a characteristic ~1.1 kb insertion in the LSC region and one rps19 deletion in the IRb repeat^29^. Noticeably, the *B. stacei* and all *B. hybridum* plastomes (D and S plastotypes) shared several insertions and common SNVs (Supplementary Fig. 3).

### Nuclear phylogenomics and genetic structure

Phylogenomic analysis was conducted with eight *B. hybridum* lines (16 subgenomes), 99 *B. distachyon* lines and one *B. stacei* line (Supplementary Data 4, Supplementary Fig. 4) using 745,858 orthologous nucleotide positions (Supplementary Data 5). These were selected because they were polymorphic (SNVs) between at least one pair of *B. distachyon* lines. Fifteen percent (114,694 of 745,858) of these were polymorphic (SNVs) between at least one pair of *B. stacei*/S subgenomes.

**Fig. 4:**
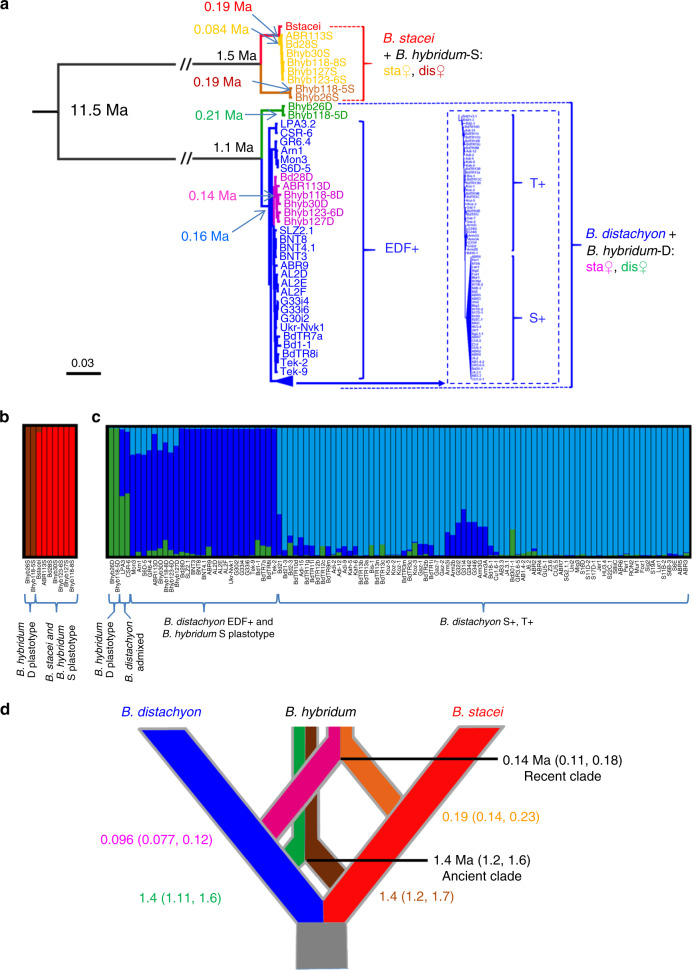
Phylogenomic analysis of nuclear genomes and dating. **a** Best maximum-likelihood *Brachypodium* phylogenomic tree based on 745,854 syntenically aligned nuclear SNVs. Most branches show strong support (Supplementary Fig. 6). Divergence times for key branches are indicated based on the tree in Supplementary Fig. 7. *B. stacei* (red), *B. distachyon* (blue), *B. hybridum* D plastotypes (S subgenome, brown; D subgenome, green), *B. hybridum-*S plastotype (S subgenome, yellow; D subgenome, purple). **b**, **c** Bayesian genomic structure analysis based on 5443 SNVs. Percentages of membership of genomes and subgenomes to the inferred genomic groups: **b** *B. stacei* and S subgenomes (*k* = 2), **c** *B. distachyon* and D subgenomes (*k* = 3). **d** Cross-bracing analysis to determine the dates of formation for the recent and ancient *B. hybridum* clades. The numbers on the diagonals represent the estimated divergence times of the D and S subgenomes from the diploid progenitor *B. distachyon* and *B. stacei* genomic lineages.

The best maximum-likelihood tree contained a main split between *B. stacei* and *B. distachyon*, and the root placement of this split was confirmed using a subset of 5443 nucleotide positions for which we could identify orthologous loci in *Oryza sativa* (Fig. 4a; Supplementary Figs. 5, 6, Supplementary Data 6). Fourteen percent of these (779 of 5443) were polymorphic (SNVs) between at least one pair of *B. stacei*/S subgenomes. As expected, the S subgenomes of *B. hybridum* grouped with *B. stacei* and the D subgenomes with the *B. distachyon* lines. The D and S subgenomes from both *B. hybridum* D-plastotype lines, Bhyb26 and Bhyb118-5, formed clades that were sisters to the rest of the D subgenomes/*B. distachyon* genomes and the rest of the S subgenomes/*B. stacei* genomes, respectively (Fig. 4a; Supplementary Fig. 6). The D subgenomes of the S-plastotype *B. hybridum* lines formed a clade nested within the extremely delayed flowering (EDF+) group of *B. distachyon* (Fig. 4a; Supplementary Fig. 6). Together this suggests that the D-plastotype lineage either formed before the radiation of the existing *B. distachyon* diversity or that the parents of the D-plastotype lineage belong to an unsampled group of *B. distachyon*. With only one *B. stacei* line included in the analysis, we cannot reach solid conclusions about the origin of the S subgenomes, though the topology of the *B. stacei* subtree also supports an ancestral *B. stacei* parent for Bhyb26 and Bhyb118-5 (Fig. 4a; Supplementary Fig. 6).

We examined genetic structure using the set of 5443 SNVs described above. The *B. stacei*/S subgenomes formed two groups with the D-plastotype lines Bhyb26 and Bhyb118-5 being genetically isolated from the others (Fig. 4b). The *B. distachyon* clade contained three groups (Fig. 4c). Interestingly, LPA3.2 and CSR-6 were admixed with nearly equal proportion of the D plastotype and EDF + SNVs, indicating that these lines are more similar to the ancestors of the D-plastotype lines than the other *B. distachyon* lines. The D-plastotype D subgenomes were genetically isolated, whereas the S-plastotype D subgenomes showed introgression with the other groups (Fig. 4c).

We dated the main splits of the *Brachypodium* tree using a coalescence model based on 4942 nucleotide positions. We calibrated the age of the *Brachypodium* crown node using a secondary constraint (normal prior mean 11.6 million years ago (Ma) SD 1.0) that encompassed previous fossil calibration-based nested dating^26,29^. Within the *B. stacei* lineage, the S subgenomes of the *B. hybridum* D-plastotype clade diverged from its ancestral *B. stacei* lineage 1.5 Ma and the S subgenomes of the *B. hybridum-*S-plastotype clade from its parental *B. stacei* lineage 0.19 Ma. Within the *B. distachyon* lineage, the D subgenomes of the *B. hybridum* D-plastotype clade diverged from its ancestral *B. distachyon* lineage 1.1 Ma and the D subgenomes of the *B. hybridum-*S-plastotype clade from its parental *B. distachyon* lineage 0.14 Ma (Fig. 4a; Supplementary Figs. 7, 8). In order to obtain a single-age estimate for the respective origins of both *B. hybridum* clades, we used a cross-bracing strategy that forces the split times of each parental genome to be contemporaneous^30^. The D-plastotype clade formed at 1.4 Ma (95% HDP 1.1–1.6), and the S-plastotype clade formed at 0.14 Ma (95% HDP 0.11–0.18) (Fig. 4d).

### Plastome phylogenomics

**Fig. 5:**
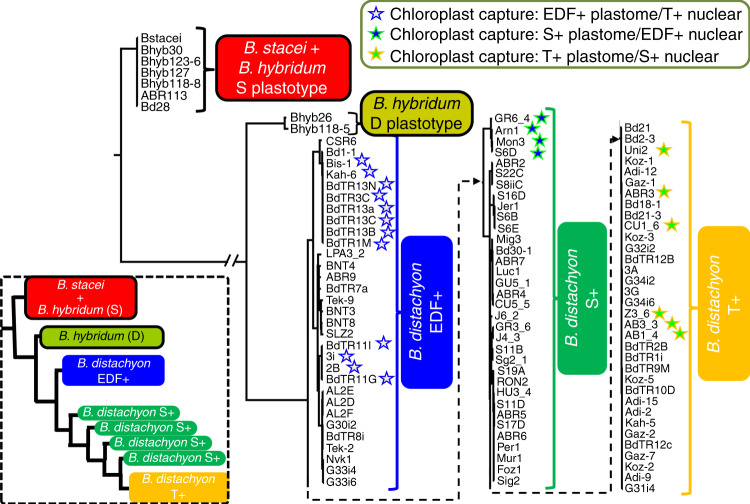
Phylogenomic analysis of plastid genomes. Best maximum-likelihood *Brachypodium* phylogenomic tree based on plastomes. Most branches of main clades show strong support (see Supplementary Fig. 2a). *B. stacei* and *B. hybridum-*S plastotypes (red); *B. hybridum* D plastotypes (olive); *B. distachyon* EDF + clade (blue); *B. distachyon* S + group (green); *B. distachyon* T + clade (yellow). Stars highlight plastid capture events between the *B. distachyon* groups (see also Supplementary Fig. 2b).

Plastome phylogenomic analysis was consistent with previous studies^29^, showing a main split for the *B. stacei* and *B. distachyon* clades (Fig. 5; Supplementary Fig. 2a). The *B. hybridum-*S-plastotype plastomes clustered with *B. stacei*, whereas the D-plastotype plastomes diverged earlier than the *B. distachyon* plastomes. Interestingly, two *B. hybridum* lines (Bhyb118-5 and Bhyb118-8) from the same location in southeastern Spain had different plastotypes (Fig. 5; Supplementary Fig. 2a). Comparison of the *B. distachyon* nuclear and plastome trees revealed multiple chloroplast capture events (some previously reported) in all three groups, indicating a history of intercrossing between groups (Fig. 5; Supplementary Fig. 2b).

### Repetitive k-mer analysis

**Fig. 6:**
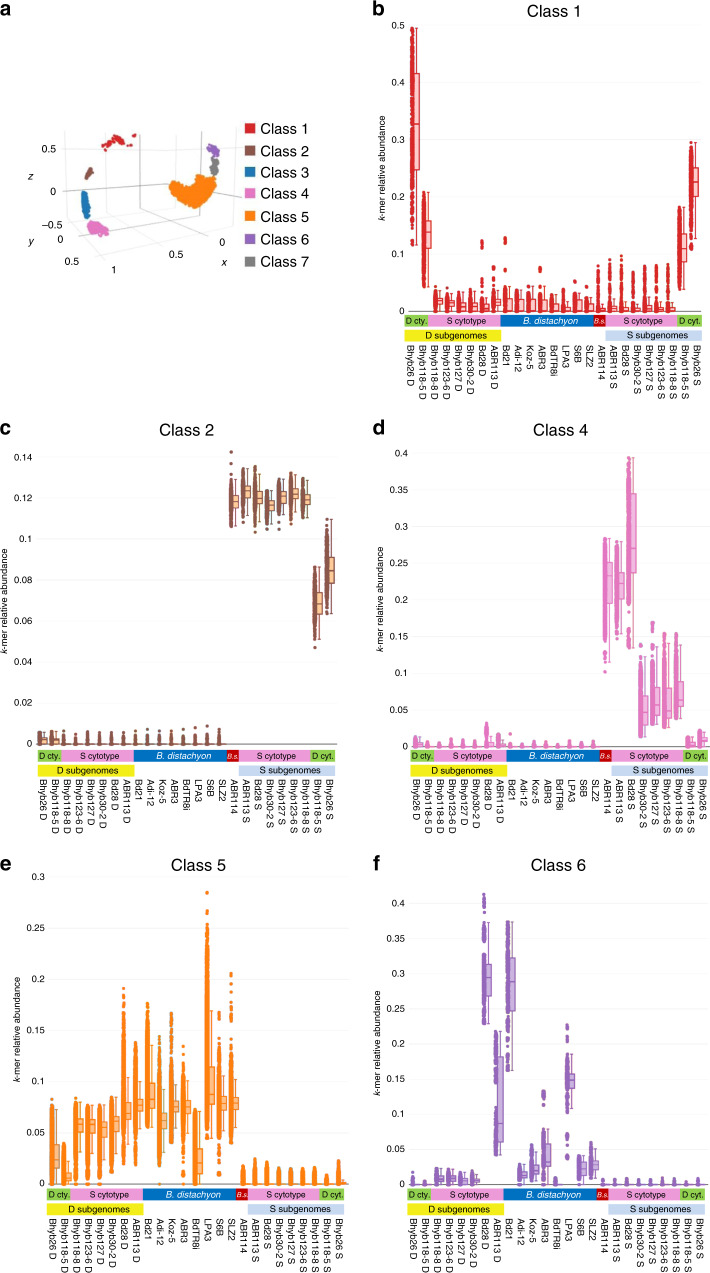
K-mer analysis. **a** Three-dimensional kernel PCA (cosine kernel) plot showing the seven distinct classes of k-mers identified by the algorithm. Note the clear separation between classes. **b**–**f** Abundance of individual k-mers in each genome/subgenome are plotted for each k-mer class designated by color. The species, subgenome, and plastotype are indicated below the *x* axis. B.s. *B. stacei*. The classes correspond to expected histories of k-mer (repeat) abundance as follows: **b** repeats that expanded after the *B. hybridum* D-plastotype lines formed, but that did not expand in the S-plastotype lines (class 1); **c** repeats that proliferated in *B. stacei*, but not *B. distachyon* before the formation of all *B. hybridum* lines examined (class 2); **d** repeats that proliferated in *B. stacei* after the formation of the D-plastotype *B. hybridum* lines, but before the divergence of the S-plastotype *B. hybridum* lines (class 4); **e** repeats that proliferated in *B. distachyon*, but not in *B. stacei* before the formation of all *B. hybridum* lines examined (class 5); **f** repeats that show differential abundance in *B. distachyon* and *B. hybridum* lines, suggesting *B. distachyon* founder effects (class 6). However, the lines with the highest abundance (Bd21, ABR113, Bd28) are the lines with very complete assemblies, so technical bias cannot be ruled out. **b**–**f** Dots represent relative abundance of individual k-mers. Boxplots show the median and 25–75% range. Error bars are + /− 1 sd. Plots for classes 3 and 7 are in Supplementary Fig. 9. Source data are provided as a Source Data file.

We studied short repetitive DNA sequences, k-mers, as a proxy for transposable elements (TEs), to look for signatures of multiple independent polyploidization events in *B. hybridum*. We counted individual 26-mers in eight *B. distachyon* genomes, the *B. stacei* genome and eight *B. hybridum* genomes, two from D-plastotype lines and six from S-plastotype lines (Supplementary Data 4). K-mers that were present in at least 100 copies in one genome with at least fivefold more copies in one pairwise comparison were fed into a machine-learning algorithm that grouped k-mers with similar enrichment patterns into seven different classes (Fig. 6a; Supplementary Fig. 9a). Remarkably, these classes fit expectations based on TE biology. Class 1 contains k-mers that are enriched in both subgenomes of the two D-plastotype *B. hybridum* lines (Fig. 6b). This pattern is consistent with TEs that proliferated into both subgenomes after the ancestral polyploidization. This enrichment is not found in the S-plastotype lines, which supports separate polyploidization events for the two plastotypes. Classes 2 and 3 (Fig. 6c; Supplementary Fig. 9b) contain k-mers that are enriched in the *B. stacei* genome and all of the S subgenomes. This is consistent with repeats that proliferated in the *B. stacei* lineage after its split from the last common ancestor with *B. distachyon*, but before the formation of both *B. hybridum* plastotypes. That the D-plastotype lines have lower abundance in both classes suggests different *B. stacei* parental lines for each plastotype. Class 4 (Fig. 6d) is similar to classes 2 and 3, but the k-mers are nearly absent from the D-plastotype S subgenomes, indicating that the proliferation in *B. stacei* may have occurred after the polyploidization event that formed the D-plastotype *B. hybridum* lines. Class 5 k-mers (Fig. 6e) are enriched in all *B. distachyon* lines and D subgenomes. This is consistent with repeats that proliferated in the *B. distachyon* lineage after its split from the common ancestor with *B. stacei* and were then passed on to *B. hybridum*. Classes 6 and 7 (Figs. 6f and Supplementary Fig. 9c) highlight the intraspecific variation of these k-mers in *B. distachyon* and *B. hybridum*. Overall, these results clearly indicate that *B. hybridum* arose at least twice, and are consistent with the nuclear and chloroplast phylogenetic trees (Figs. 4 and 5). In addition, the distinct k-mer patterns observed in the D and S-plastotype lines (Fig. 6b, d) indicate that there has been little mixing between these groups.

**Fig. 7:**
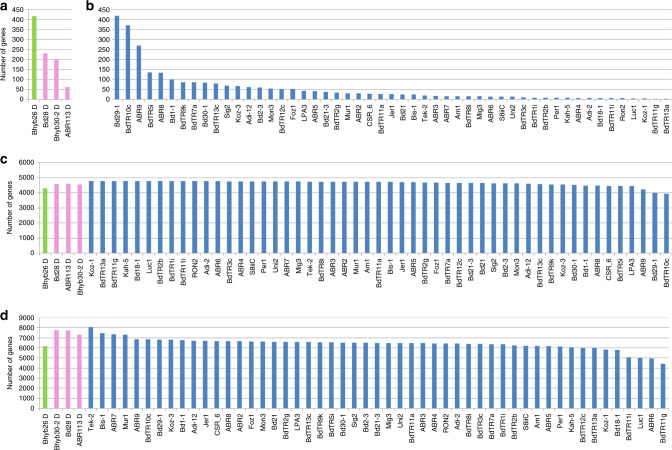
Comparative pan-genomic analysis. Comparison of the *B. hybridum* D subgenomes to the *B. distachyon* pan-genome. **a** The number of genes that were found in all 52 *B. distachyon* lines (core genes with respect to *B. distachyon*) that were missing from each D subgenome. **b** The number of genes that were missing from each respective *B. distachyon* line but present in all other lines. These genes would have been core genes if that particular line had not been used to create the pan-genome. **c** The number of soft-core genes (found in 54–52 of all genomes/subgenomes) in each line. **d** The number of shell genes (found in 51-3 of all genomes/subgenomes) in each line. The *B. hybridum* D-plastotype Bhyb26 line is indicated in green, the *B. hybridum-*S plastotype ABR113, Bhyb30, and Bd28 lines in pink, and the *B. distachyon* genomes in blue. Note that the *B. hybridum* D subgenomes values fall within the *B. distachyon* genomes values. Source data are provided as a Source Data file.

### Crossing ancestral and recent *B. hybridum* lines

Multiple lines of evidence suggest that the *B. hybridum* D and S plastotypes are reproductively isolated. We experimentally tested their compatibility using controlled crosses and found that the D and S plastotypes are incompatible (Supplementary Fig. 10; Supplementary Note 1).

### Pan-genomic analysis

Previous analysis of the *B. distachyon* pan-genome revealed extensive presence/absence variation^15^. Thus, simply comparing a polyploid genome to single reference genomes from the progenitor species will greatly overestimate the changes that occurred after polyploidization. In order to differentiate changes that occurred after polyploidization from intraspecific differences in the progenitors, we conducted a pan-genomic analysis. We separated the D and S subgenomes of five *B. hybridum* lines and included them in a pan-genome analysis together with 51 *B. distachyon* lines and *B. stacei* (Supplementary Data 4). The coding sequence of genes (CDS) was clustered using the same method used to create the *B. distachyon* pan-genome^15^. The resulting matrix of gene clusters vs. lines (Supplementary Data 7 and 8) was queried to compare the variation in the *B. hybridum* D subgenomes to the variation among *B. distachyon* genomes. Overall, the D subgenomes looked like typical *B. distachyon* genomes in terms of the proportions of shell, soft-core and core genes (Fig. 7). We did note that the D subgenome from the *B. hybridum* D-plastotype line Bhyb26 was missing more *B. distachyon* core genes (genes found in all *B. distachyon* lines) than the other *B. hybridum* lines. To compare this to the intraspecific variation in *B. distachyon*, we determined how many pan-genes in each *B. distachyon* line were missing from only that line. Since these genes would have been core genes if the pan-genome was constructed without that particular line, this is the appropriate comparison with the number of *B. distachyon* core genes missing from the *B. hybridum* D subgenomes. As the number of genes missing from only one *B. distachyon* line was similar to the number of core genes missing from the *B. hybridum* D subgenomes, the core genes missing from the *B. hybridum* D subgenomes as a whole could simply have been missing from the actual genomes of the *B. distachyon* parents of the polyploids (Fig. 7).

### Selection analysis

Potential selection pressure effects were investigated in five *B. hybridum* lines, *B. stacei* and 44 *B. distachyon* lines (Supplementary Data 4) using genes present in all lines as determined by the pan-genome analysis. Selection rates were estimated based on the dN/dS ratio over the full length of the CDSs in two groups of genes: one containing the *B. distachyon* lines and the *B. hybridum* D subgenomes genes (D group) and the other containing *B. stacei* and the *B. hybridum-*S subgenomes genes (S group). In order to avoid phylogenetic bias, pairwise dN/dS comparisons were computed with respect to an outgroup: *B. stacei* for the D group and *B. distachyon* for the S group. Our results indicated similar levels of selection pressure on genes in the *B. hybridum* subgenomes and in the corresponding progenitor species genomes (Supplementary Fig. 11). Thus, we see no evidence of a release from negative selection pressure in the genes of the polyploids.

### Reference-based analysis

**Fig. 8:**
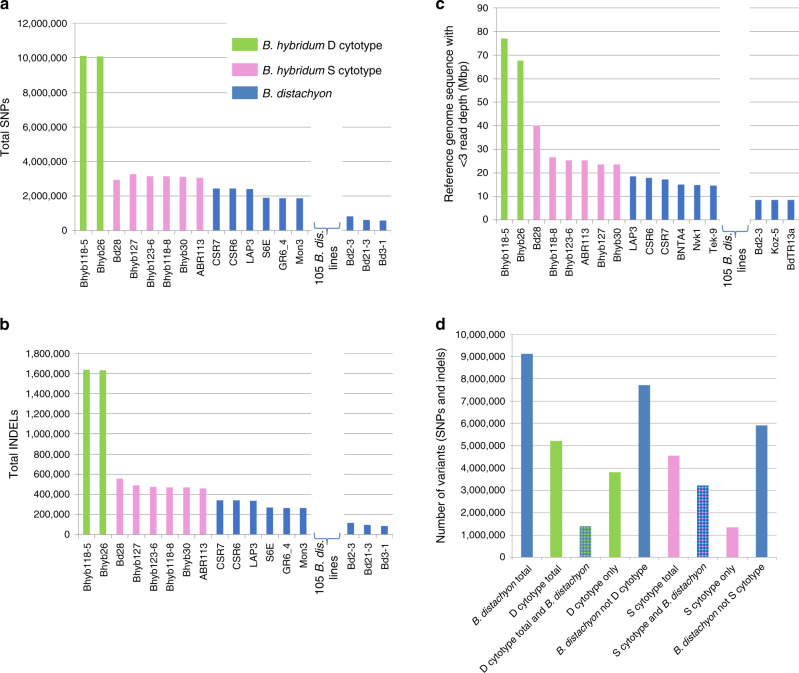
Reference-based analysis. Individual Illumina sequence reads, or simulated reads created by shredding a PacBio assembly (Bd28 accession), were mapped to the *B. distachyon* Bd21 reference genome to identify SNVs (**a**), small indels (**b**), and larger missing or highly divergent regions (**c**). In each graph, the lines have been ordered by descending number of polymorphisms, showing a trend from high levels in the *B. hybridum* D-plastotype lines, moderate levels in the *B. hybridum-*S-plastotype lines, and low levels in the *B. distachyon* lines. For clarity, 105 intermediate *B. distachyon* lines were omitted from the graphs. The *B. hybridum* D and S-plastotype lines both have significantly more SNVs, indels and deleted or highly divergent regions than *B. distachyon* (*t* tests, *P* < 10^−20^), whereas the *B. hybridum* D-plastotype lines had significantly more SNVs, indels (*t* test, *P* < 10^−9^) and deletions or highly divergent regions (*t* test, *P* < 10^−4^) than the S-plastotype lines. Note that the amount of deletion/highly divergent regions in Bd28 may be inflated by using a PacBio assembly that has more repetitive sequence assigned to the D subgenome than is possible by mapping Illumina reads. **d** The number of variants shared and unique to different groups. Green bars correspond to *B. hybridum* D plastotype, pink bars to *B. hybridum-*S plastotypes, and blue bars to *B. distachyon* accessions. Source data are provided as a Source Data file.

We conducted a reference-based analysis of the D subgenomes from eight *B. hybridum* lines and 114 *B. distachyon* lines (Supplementary Data 4) to examine the spectrum of smaller structural changes (SNVs and small indels) and larger deletions and/or areas of high divergence relative to the *B. distachyon* Bd21 v3.1 reference genome. The analyses indicated that the *B. hybridum* D-plastotype lines were more divergent from *B. distachyon* Bd21 than the S-plastotype lines (Fig. 8). However, the S-plastotype D subgenomes were also significantly different (*t* test, *P*-value < 10^−20^) from the *B. distachyon* lines, indicating that while these D subgenomes are very similar to the *B. distachyon* genomes, they have accumulated numerous changes since the formation of the polyploids (Fig. 8).

Interestingly, the *B. distachyon* lines that were closest to the D-plastotype lines in the phylogenetic tree and the structure analysis (Fig. 4a, c) were also the most divergent from the reference *B. distachyon* Bd21 genome. The majority (73%) of the SNVs in the two *B. hybridum* D-plastotype lines were not found in any *B. distachyon* line, whereas the converse was true for the six *B. hybridum-*S-plastotype lines, where only 30% of the variants were not shared with *B distachyon*, despite the fact that a larger number of S-plastotype lines were analyzed (Fig. 8d). This is consistent with a model in which the D-plastotype lines diverged earlier than the S-plastotype lines and over time accumulated novel mutations not found in *B. distachyon*.

### Expression analysis and pseudogenes

Many polyploids exhibit homeolog-expression bias, where homeologs from one subgenome are systematically more highly expressed than homeologs from the other subgenome^31,32^. RNA-seq in *B. hybridum* ABR113 leaves and spikes revealed that the distribution of gene expression values was very similar in each subgenome (Supplementary Note 2; Supplementary Fig. 12; Supplementary Data 9).

The *B. hybridum* genome was scanned for pseudogenes, which are expected to accumulate in polyploids due to the relaxed natural selection that is believed to accompany genome duplication^32^. We examined regions of the *B. hybridum* genome where no gene was annotated, but where a gene was expected to exist based on synteny with the diploids. Only 24 candidate pseudogenes were identified. Twenty-two of the 24 pseudogenes were from the D subgenome. While this overrepresentation of D subgenome pseudogenes is intriguing, more study is needed to determine its biological significance. The dearth of high-confidence pseudogenes in *B. hybridum* ABR113 is consistent with our pan-genomic analysis.

## Discussion

Multiple lines of evidence indicate that *B. hybridum* arose from at least two distinct polyploidization events (Figs. 4–6). This agrees with the fact that the D and S plastotypes were formed from reciprocal crosses between diploid progenitors (Fig. 1). Surprisingly, our k-mer, STRUCTURE, and phylogenetic analysis all indicate very little or no historical crossbreeding between these groups (Figs. 4 and 6). This was particularly striking for Bhyb118-5 and Bhyb118-8 because they were collected from the same location. The incompatibility between D and S-plastotype lines in controlled crosses explains this genetic isolation. Thus, the different *B. hybridum* plastotypes may be cryptic species.

Dating analysis revealed that the S-plastotype lines formed ~0.14 Ma, and that the D-plastotype lines formed ~1.4 Ma. The very young ages of the S-plastotype lines (Fig. 4d) is consistent with the nested positions of the S and D subgenomes within the respective *B. stacei* and *B. distachyon* clades and their almost contemporary ages with subgroups within *B. stacei* (0.19 Ma) and *B. distachyon* (0.096 Ma; Fig. 4a; Supplementary Fig. 7). By contrast, the D-plastotype lines formed ~1.4 Ma (Fig. 4d) and the sister position of its subgenomes in the *B. distachyon* and *B. stacei* lineages supports the hypothesis of preferential survival of genomes in better adapted allopolyploids than in their potentially extinct diploid parents^33^. It is also possible that we have simply not sampled populations of *B. distachyon* and *B. stacei* lines that are similar to the parents of the D-plastotype lines.

We found insertions of portions of the S plastome into the D plastome that could have resulted from heteroplasmy and recombination between ancestral D and S plastomes in the *B. hybridum* D-plastotype lines (Supplementary Fig. 3). This is consistent with previous reports of heteroplasmy and plastid recombination in *B. distachyon*^29^. While multiple founder events based on different plastotypes have been proposed for other allopolyploids, such as *Arabidopsis kamchatica*^34^, plastid heteroplasmy has rarely been reported^35^.

We observed no large structural variation between the *B. hybridum* ABR113 genome and the diploid reference genomes *B. distachyon* Bd21 and *B. stacei* ABR114 both by overall synteny (Fig. 2a, b) and by cytomolecular characterization (Fig. 3; Supplementary Fig. 1). This is similar to the high synteny observed between wheat subgenomes and may simply be due to the relatively recent formation of these polyploids^36^. However, since we did not have a highly contiguous assembly to evaluate synteny in a *B. hybridum* D-plastotype line, we do not know the extent of large structural rearrangements in this older lineage. Our pan-genomic analysis identified thousands of differentially present genes between the reference *B. distachyon* genome and the *B. hybridum* D subgenomes, however, the vast majority of this genic presence/absence variation was also present in at least some of the *B. distachyon* lines (Fig. 7; Supplementary Data 7). However, we cannot say that the same gene deletion/creation event is responsible for the loss of orthologous genes in any two lines at this time. Thus, there is no support for large-scale gene loss after polyploidization. This differs from the observation of some polyploid plant genomes where extensive genomic rearrangements and gene losses have been observed rapidly after polyploidization, especially in newly created *Brassica*, *Arabidopsis* and wheat allopolyploids^37^, but is similar to other allopolyploid plants with more stable genomes, such as synthetic allotetraploid cottons and wild allopolyploid *Spartina anglica*^37,38^. Contrary to general expectations^39^, we did not detect significant overall differences in selection pressure on polyploid genes indicating that even after 1.4 million years homeologous genes face a similar selection pressure (Supplementary Fig. 11). This is unlike the relaxation of purifying selection that was observed in the orthologous genes of the allohexaploid wheat genomes compared to those of their diploid parental genomes^40^. However, the wheat polyploidization events are much older^41^ than *B. hybridum*. We did observe large numbers of SNVs and small indels in the D-plastotype D subgenomes and a lesser, but highly significant, amount of polymorphism in the S-plastotype D subgenomes with respect to the *B. distachyon* reference genome (Fig. 8); based on k-mer distribution, we also found evidence for the movement of TEs between the D and S subgenomes in the old polyploids (Fig. 6b).

We did not find any sign of genome dominance (an excess of homeolog-expression bias from one subgenome) in leaves and spikes. Genome dominance is typically correlated with the loss of sequence (fractionation) in the non-dominant genome(s)^32,42^. The genomic stability and equal transcriptomic contributions in the *B. hybridum* ABR113 subgenomes indicate that there is no sign of fractionation in this genome. This puts *B. hybridum* ABR113 in a class with several allopolyploids, which are structurally stable and show only subtle or no genome dominance^43,44^.

Taken together, our data support a scenario where the genes of *B. hybridum* evolve slowly after polyploidization with only small changes evident after a few hundred thousand years and a sizeable number of small polymorphisms evident after one million years, but no large amount of gene loss even after 1.4 million years. We did see evidence (k-mer profiles and lack of read mapping to sections of the reference genome) that repetitive DNA and other noncoding DNA is changing more rapidly than genes. Our results also highlight that allopolyploid species may in fact be composed of independent reproductively isolated clades formed at different times from different progenitors. This is consistent with other studies of wild allopolyploids where this may be the rule rather than the exception^45,46^. *Brachypodium hybridum* is an extreme case because young and old clades occupy the same geographical site, yet remain genetically distinct.

The gradual changes we observed after polyploidization differ from many previous studies. However, it should be noted that we would have reached very different conclusions had we compared a single polyploid genome to single-progenitor diploid genomes. Only by taking a pan-genomic approach could we discern that the variation within the allopolyploid subgenomes was well within the intraspecific variation of at least one of the parents rather than a consequence of polyploidy per se.

## Methods

### Plant germplasm and sequencing

The lines used in this study and their sources are described in Supplementary Data 4, and a map of their origins is shown in Supplementary Fig. 4. High-molecular-weight nuclear genomic DNA was isolated from 10–20 g of leaf tissue collected from 4-week-old seedlings and immediately frozen in liquid nitrogen. Tissue was finely ground into powder with mortar and pestle. DNA was isolated via a nuclei isolation protocol^15^. First, frozen powdered tissue was resuspended by gentle swirling on ice in 250-mL beakers with sucrose extraction buffer containing beta-mercaptoethanol over a period of 20 min. Nuclei were filtered through a wide mesh cheese cloth to filter larger tissue fragments and further purified through multiple rounds of centrifugation in high-percentage sucrose buffer and subsequent decanting of supernatant. DNA was released to solution by digestion with Proteinase K followed by incubation with SDS. DNA was purified by multiple rounds of phenol:chloroform:isoamyl purifications followed by centrifugation until a clear aqueous/organic interface was observed. DNA was precipitated through addition of sodium acetate followed by an equal volume of isopropanol in microcentrifuge tubes. After 30-min incubation on ice, tubes were centrifuged at 4 °C for 30 min at 19,000 *g* in a tabletop microcentrifuge. Resulting pellets were washed by three rounds of 1 mL of ice-cold ethanol followed by centrifugation and decanting of the supernatant. Final pellets were briefly air-dried at room temperature and then resuspended in TE. Resulting DNA was prepared into library according to the manufacturer’s instructions. PacBio sequencing was performed on a PacBio RSII instrument. For Illumina sequencing, DNA was randomly sheared into desired fragment sizes, and then used to create Illumina fragment libraries or alternatively DNA was prepared according to the appropriate Nextera mate pair library protocol. Illumina sequencing was performed on Illumina HiSeq2500 and Illumina MiSeq sequencers at the Joint Genome Institute.

### Genetic mapping populations

Genetic mapping populations were constructed for both *B. hybridum* and *B. stacei*. Lines ABR113 and BdTR6g of *B. hybridum* and ABR114 and TE4.3 of *B. stacei* were, respectively, crossed, and the F1 hybrids were confirmed using PCR markers. DNA was extracted from 200 F2 individuals from a single F1 hybrid in each case. Illumina fragment libraries were constructed for 192 samples for each mapping population. The *B. hybridum* and *B. stacei* populations were sequenced to 7× and 4× average coverage, respectively. For *B. stacei* 174 samples and for *B. hybridum* 167 F2 lines were sequenced to sufficient depth, and free from contamination to be included in the final genetic maps. Parental lines of respective mapping populations were sequenced to greater than 50× depth and non-repetitive 51-mers, in which the first 50 nucleotides were identical between both parents, and the last base contained a SNV distinguishing the parents were identified. Raw genotypes of individuals within mapping populations were determined by matching k-mers from the whole-genome skim sequencing of individuals to the markers described above. A given marker locus was determined as either homozygous for one of the parents if k-mers from only one of the respective parents was observed, or alternatively heterozygous if alleles for both parents were observed at a given marker locus. Raw genotypes were further processed into consensus genotypes for 7–14-kb fixed intervals across the draft assembly scaffolds by a simple majority rule algorithm amongst individual SNVs within each interval that required a given parental genotype to be observed at twice the frequency of the alternate parental allele and also twice the frequency of heterozygous calls. Likewise, a heterozygous consensus call was made if heterozygous genotypes were twice the frequency of both parental genotypes. Otherwise the consensus genotype was undetermined (Supplementary Data 2 and 3).

### Genome assembly

The *B. stacei* reference genome was created using the ALLPATHS (vLG)^47^ assembler with 204× depth of Illumina reads from a combination of short fragment libraries and long-range mate pair libraries (1.5 kb, 4 kb, 7.5 kb). To validate and order scaffolds, a 22,043 marker genetic map was used. Assembly errors were identified by looking for simultaneous changes in haplotype across most F2 lines in each scaffold (Supplementary Data 2). A total of six breaks were made in the original scaffolds. The scaffolds were then oriented, ordered, and joined together into ten chromosomes using a genetic linkage map produced by MST map (v1.0.0-2015)^48^ from all 22,043 consensus markers. A total of 554 joins were made during this process.

Initial assembly of *B. hybridum* reference genome from inbred line ABR113 was performed using the Meraculous assembler (2.2.2.5 release) in diploid mode 2 in order to prevent collapse of subgenome sequences. The assembly utilized 28× of Illumina sequence from 250-bp paired-end sequences generated from an 800 bp-insert fragment-library and three long-range mate pair libraries (1 kb, 4 kb, and 7 kb). A genetic map was used to verify scaffolds as described for *B. stacei*, and two scaffolds were broken. The map was also used to order and orient the scaffolds to produce an interim assembly. The interim assembly was improved using additional Illumina sequence from Dovetail Genomic’s Chicago^TM^ library and the HiRise^TM^ assembler^49^. During this process, 17 scaffolds were broken, 825 joins were made, and 64 gaps closed. The resulting scaffolds were essentially chromosome arms with a remarkable 15 Mb N50. The scaffolds were then oriented, ordered, and joined together into 15 chromosomes (504 Mb) using a genetic linkage map produced by MST map (v1.0.0-2015) using all consensus markers (Supplementary Data 3). Final scaffolds were gap-filled yielding a final set of 8327 contigs within the 15 chromosomes (main genome contig N/L50: 1,128/135.1 kb). The final assembly was compared with the genetic map to verify order and orientation of sequences along the chromosomes.

*Brachypodium distachyon* lines were assembled with the massively parallel HipMer genome assembler (v0.9.6)^50^ on the Edison super-computer at the National Energy Research Scientific Computing Center, Lawrence Berkeley National Laboratory (USA). ALLMAPS (v0.7.5)^51^ was used to order and orientate scaffolds based on synteny to the reference *B. distachyon* Bd21 genome.

*Brachypodium hybridum* lines Bhyb30, Bhyb26, Bhyb118-5, Bhyb118-8, Bhyb123-6, and Bhyb127 were assembled from ~60× depth of Illumina paired-end reads using Meraculous assembler (2.2.2.5 release) as explained above. These assemblies were nearly complete, but fragmented (Supplementary Data 1). Assembled scaffolds were ordered and orientated by alignment to the *B. hybridum* chromosome-level assembly of ABR113.

### Annotation

*Brachypodium hybridum* lines ABR113, Bd28, Bhyb30, Bhyb26, and Bhyb118-5 as well as the *B. stacei* ABR114 reference genome were annotated using the JGI annotation pipeline. EST assemblies were constructed from paired-end Illumina RNA-seq reads using PERTRAN (JGI/Phytozome internal pipeline) and PASA (v2.3)^52^. Loci were determined by transcript assembly alignments and/or EXONERATE (v2.4.0) alignments of proteins from *B. distachyon, B. stacei, A. thaliana*, rice, sorghum, foxtail, grape, soybean, and Swiss-Prot eukaryote proteins to the respective genome sequence, soft-repeatmasked using RepeatMasker (v4.0.5) (http://www.repeatmasker.org), with up to 2-kb extension on both ends unless extending into another locus on the same strand. Gene models were predicted by homology-based predictors, FGENESH + (v2.0)^53^, FGENESH_EST (v2.6) (similar to FGENESH+, EST as splice site and intron input instead of protein/translated ORF), and GenomeScan (v1.0)^54^. The highest scoring predictions for each locus were selected using multiple positive factors including EST and protein support, and one negative factor overlap with repeats. The selected gene predictions were improved by PASA. Improvement included adding UTRs, splicing correction, and alternative transcripts. PASA-improved gene model proteins were subject to protein homology analysis to the above mentioned proteomes to obtain Cscore and protein coverage. Cscore is a protein BLASTP score ratio to MBH (mutual best hit) BLASTP score and protein coverage is highest percentage of protein aligned to the best of homologs. PASA-improved transcripts were filtered based on Cscore, protein coverage, EST coverage, and its CDS overlapping with repeats. The transcripts were selected if their Cscore values were larger than or equal to 0.5 and protein coverage values larger than or equal to 0.5, or if they had EST coverage, but their CDS overlapping with repeats were less than 20%. For gene models whose CDS overlapped with repeats for >20%, their Cscore values had to be at least 0.9 and homology coverage values of at least 70% to be selected. The selected gene models were subject to Pfam analysis, and gene models whose protein were more than 30% in Pfam TE domains were removed.

RNA-seq data (Supplementary Data 9) from *B. stacei* ABR114 and *B. hybridum* ABR113 and Bd28 inbred lines were used to aid the annotation of their respective genomes. ABR113 RNA-seq data were also used to aid the annotation of Bhyb26, Bhyb30, and Bhyb118-5 lines. A total of 55,011 transcript assemblies were produced by PASA, and were used for the annotation of the *B. stacei* ABR114 genome. PASA transcript assemblies for ABR113 (199,500 transcript assemblies), Bhyb26 (124,003), Bhyb30 (126,716), Bd28 (111,639), and Bhyb118-5 (97,870) were also used for structural annotation of their respective genomes (Supplementary Data 1).

### Synteny analysis

A phylogenetic tree for 54 *B. distachyon*, one *B. stacei*, and eight *B. hybridum* lines was constructed by running IQTREE v. 1.6.7 on the concatenation of Progressive Cactus multiple alignment of sequences for 100 bootstraps. Pairwise syntenic blocks were found via an internal JGI pipeline between all of the 99 *B. distachyon* and the eight *B. hybridum* D subgenomes and eight *B. hybridum-*S subgenomes along with *B. stacei* (ABR114). Syntenic blocks were excluded if they had less than four syntenic loci. Pairwise syntenic blocks were merged together via *B. stacei* coordinates if they were within 300 kb. Sequences were extracted from each genome for the respective syntenic block, converted into multi-fasta files, and were formatted to run a multiple sequence alignment via Progressive Cactus (v0.1)^55^.

A separate Cactus whole-genome multiple sequence alignment of syntenic blocks that included rice, *B. distachyon*, and *B. stacei* was performed, and processed as described above. This is subsequently described as the outgroup whole-genome alignment.

### Karyotype analysis

Cytomolecular mapping was done using comparative chromosome barcoding (CCB) on the following genotypes of *B. distachyon* (Bd21), *B. stacei* (ABR114, Bsta5 and ABR200), and *B. hybridum* (ABR113, ABR100 and ABR117) with 80 low-repeat *B. distachyon*-derived Bacterial Artificial Chromosome clones (BACs) (Supplementary Table 1). All BACs originated from the BD_ABa and BD_CBa genomic DNA libraries derived from the fingerprinted contigs, which had previously been assigned to the respective reference chromosomes of *B. distachyon* Bd21^56^. Chromosome preparation and fluorescence in situ hybridization followed the protocol described by Jenkins et al.^57^ with minor modification specific for the CCB approach performed according to Lusinska et al.^28^. Young seedlings were incubated in a box with ice for 24 h, then fixed in 3:1 (v/v) 100% methanol/glacial acetic acid and stored at −20 °C until used. Excised root tips were washed three times for 5 min in 0.01 M citrate buffer (pH 4.8) to remove the fixative. Root tips of *B. distachyon* and *B. hybridum* were digested in an enzyme mixture consisting of 8% (v/v) pectinase (Sigma), 1% (w/v) cellulase (Sigma), and 1% (w/v) cellulase Onozuka R-10 (Serva) in 0.01 M citrate buffer for 2 h at 37 °C. For *B. stacei*, the enzyme concentrations were 6%, 0.5%, and 0.5%, respectively, with the digestion time of 2 h 40 min at 37 °C. The meristems of each species were dissected in a small volume of 45% acetic acid followed by separate mounting of the digested material on a slide and squashed in drops of acetic acid. Squashed chromosome preparations were frozen thoroughly on dry ice followed by cover slip removal and allowed to air-dry.

BAC DNAs was isolated using the standard alkaline lysis method, and labeled by nick-translation mix with tetramethylrhodamine-5-dUTP, digoxigenin-11-dUTP, or biotin-16-dUTP (all Roche). Chromosome preparations were pre-treated with 100 μg/ml RNase A in 2 × SSC buffer for 1 h at 37 °C. Then, slides were washed three times for 5 min each in 2 × SSC buffer at room temperature (RT), fixed in freshly prepared 1% formaldehyde in 1 × PBS buffer for 10 min, washed three times for 5 min each in 2 × SSC, dehydrated in 70%, 90%, and 100% ethanol series and air-dried. For CCB, the hybridization mixture consisted of 40% deionized formamide, 15% dextran sulfate, 2 × SSC, 0.5% SDS, and 50–100 ng/ml of each DNA probe. Hybridization mix was denatured at 80 °C for 10 min, plunged into ice for 10 min and applied 40 µl to each slide. The slides were covered with plastic cover slips, and denatured in a humidity chamber at 70 °C for 4.5 min followed by incubation for ~40 h in a hermetically sealed humid chamber at 37 °C. Post-hybridization washes were equivalent to ~60% stringency. In brief, slides were washed twice in 20% (v/v) formamide in 2 × SSC for 5 min at 37 °C, rinsed three times for 5 min in 2 × SSC buffer at 37 °C followed by three changes of 2 × SSC, 5 min each, at RT. For immunodetection of digoxigenin- and biotin-labeled probes, slides were washed for 5 min at RT in 0.2% (v/v) Tween20 in 4 × SSC. Blocking reagent (5% solution of nonfat dry milk in 4 × SSC) was applied to each slide followed by incubation for 30 min at RT. Then, slides were drained and 40 µl of FITC-conjugated anti-digoxigenin antibodies (Roche) and Alexa Fluor 647-conjugated anti-biotin antibodies (Jackson ImmunoResearch) diluted 1:11 and 1:100, respectively, in blocking reagent were added to each slide, followed by incubation in a humid chamber for 2 h at 37 °C. Finally, slides were washed three times for 10 min each in Tween20/4 × SSC at 37 °C and dehydrated in ethanol series. Air-dried slides were mounted and counterstained in Vectashield (Vector Laboratories) containing 2.5 µg/ml of DAPI (4′,6-diamidino-2-phenylindole; Serva). Photomicrographs were acquired using an AxioImager.Z.2 (Zeiss) wide-field epifluorescence microscope equipped with high-sensitivity monochromatic camera (AxioCam Mrm; Zeiss) followed by standard digital processing using ZEN 2.3 Pro (Zeiss) and Photoshop CS3 (Adobe).

### Plastome analysis

Plastomes of 65 newly sequenced *B. hybridum* and *B. distachyon* lines were assembled from the total WGS data using NOVOPlasty v.2.7.1^58^. The complete and fully annotated plastomes of parental diploid species *B. distachyon* ABR6 (LT222229) and *B. stacei* ABR114 (NC_036837; LT558589) were used as seeds for the assembly of the respective *B. distachyon*-type and *B. stacei*-type plastomes. All the new plastomes were assembled using a k-mer length of 39, insert size of 300 bp, and Illumina paired-end reads of 151 bp, except for *B. hybridum* Bd28 which was assembled from PacBio sequences using blasr v.5.3.2^59^, samtools v.1.4.1^60^, and Canu v.1.7.1^61^ with corrected ErrorRate = 0.105 and batMemory = 50 parameters using the *B. stacei* ABR114 plastome as a reference. Assembled plastomes were checked by visual inspection of read mappings using IGV v.2.3.8^62^. The newly assembled plastomes of *B. distachyon* and *B. hybridum-*S plastotypes were annotated using the *B. distachyon* ABR6 and *B. stacei* ABR114 annotations as reference as described by Vu et al.^45^. The plastome of *B. hybridum* Bhyb26 (D plastotype) was annotated using cpGAVAS^63^ and curated to be used as reference for annotation of the Bhyb118-5 plastome. The newly annotated plastomes of *B. distachyon* and *B. hybridum* ecotypes were deposited at ENA (European Nucleotide Archive; https://www.ebi.ac.uk/ena) with accessions numbers LR537446 – LR537510 (Supplementary Data 4).

A multiple alignment of the newly assembled plastomes and of three previously annotated plastomes of *B. distachyon*, *B. stacei*, and maternal stacei-type *B. hybridum* plastomes^45^ was performed with MAFFT v.7.215^64^. The multiple plastome alignment was filtered to remove poorly aligned regions through the automated option of trimAl v.1.2rev59 software^65^ (Supplementary Data 10).

Noticeably, the *B. hybridum* D-plastotype plastomes share six insertions and common SNVs in two plastid regions in their predominantly *B. distachyon*-type plastid genomes with the *B. hybridum-*S plastotypes and *B. stacei* plastomes (Supplementary Fig. 3). These plastid introgressions of the S plastome into the D plastome in Bhyb26 and Bhyb118-5 were probably ancient as none of the nuclear S and D subgenomes of the studied *B. hybridum* lines show evidence of introgressions between the old and young clades or backcrosses of the old hybrids to the *B. stacei* parent (Fig. 4b).

### Phylogenomic and genomic structure analysis

In total, 745,854 SNVs were extracted from the multisequence alignment of in-group genomes (Supplementary Data 5), described in the above synteny analysis section, into VCF format using Maffilter (v1.3)^66^ and MafStrander (v1.0) (https://github.com/dentearl/mafTools/tree/master/mafStrander). The VCF file was then converted into a multi-fasta and a final nexus file used in the nuclear phylogenomic analysis of in-group taxa only. This SNV set was intersected and merged with SNVs from the outgroup whole-genome alignment, yielding a final 5443 SNV VCF file that was converted into a multi-fasta and a final nexus file used in the nuclear phylogenomic analysis of in-group plus *Oryza sativa* as an outgroup (Supplementary Data 6).

A nuclear maximum-likelihood (ML) optimal tree based on the 745,854 syntenic SNVs, from 116 genomes/subgenomes (99 *B. distachyon* genomes, 1 *B. stacei* genome, and 16 *B. hybridum* subgenomes), was constructed with IQTREE v. 1.6.7^67^. The best-fit evolutionary model (TN + F + ASC + R6) was automatically selected by the ModelFinder option^68^ in IQTREE based on the Bayesian information criterion (BIC). The program computed 20 ML starting trees from 98 alternative randomized maximum parsimony (MP) trees, searching for best-scoring ML trees and estimating branch support for the best tree from 1000 bootstrap replicates using the ultrafast bootstrap option^69^. Similarly, an optimal plastid ML tree based on the 108 aligned plastomes (99 from *B. distachyon*, one from B*. stacei* and eight from B. *hybridum*) based on 137,321 homologous plastid DNA positions (1782 parsimony-informative) was computed with IQTREE v. 1.6.7 using the same parameters as in the nuclear search and after inferring TVM + F + R4 as the best-fit evolutionary model for the plastid data. Both *Brachypodium* plastome and nuclear SNV trees were mid-point rooted, as this position was similar to that retrieved when using *O. sativa* to root the nuclear 5443 SNV-based topology (Supplementary Fig. 5). The best nuclear and plastome ML trees were visualized with FigTree (v. 1.4.0) (http://tree.bio.ed.ac.uk/software/figtree/).

Nuclear genomic structure among the highly divergent *B. stacei*-type (*B. stacei* plus *B. hybridum-*S subgenomes) and *B. distachyon*-type (*B. distachyon* plus *B. hybridum* D subgenomes) lines was assessed with STRUCTURE (v. 2.3.4), imposing in each separate analysis an admixture ancestry model and a correlated allele frequencies model. We estimated values of genomic-group differentiation (K) between 1 and 4 for the *B. stacei*-type (S) group and between 1 and 7 for the *B. distachyon*-type (D) group, considering that up to two and up to five main genetic groups were detected respectively for each lineage in our phylogenetic ML analysis (see Fig. 4a). In all cases, each search consisted of an initial burn-in of 10,000 Markov Chain Monte Carlo (MCMC) iterations followed by 100,000 additional MCMC iterations, and estimation of cluster membership (q) set to a 10% threshold value. Ten replicates were run for each K. The number of genomic groups (clusters) in the data was estimated using STRUCTURE HARVESTER (v. 0.9.94)^70^, which identifies the optimal *K* based both on the posterior probability of the data for a given K and the rate of change in the likelihood distribution among Ks (∆K)^71^. The graphical outcomes were visualized using the software DISTRUCT (v. 1.1)^72^.

### Dating and cross-bracing analysis

Ancestral split times of the *B. stacei* and homeologous *B. hybridum-*S-type subgenomes’ groups and of the *B. distachyon* lineages and homeologous *B. hybridum* D-type subgenomes’ groups were estimated from a reduced nuclear dataset of 4942 SNVs and 41 tips representing all subgenomes of ancestral and recent *B. hybridum* plastotypes, *B. stacei*, the *B. distachyon* EDF + and earlier clades and selected clade members of the *B. distachyon* T + and S + groups (Supplementary Data 4) using the SNAPP package of the BEAST 2.4.7 software^73^. We imposed several prior distributions: 1/x for the clock rate and lambda (Yule model), a uniform distribution for theta, and a normal distribution for a secondary age constrain at the *Brachypodium* crown node (mean = 11.6 Ma, SD = 1.0). The forward and backward mutation rates were fixed to give one expected mutation per unit of time. We ran 2,410,000 MCMC generations in SNAPP with a sampling frequency of 1000 generations, discarding the first 10% steps as burn-in. The adequacy of parameters was checked using TRACER v.1.6 (http://beast.bio.ed.ac.uk/Tracer) with most parameters showing Effective Sample Size (ESS) > 200. A maximum clade credibility tree was computed after discarding 10% of the respective saved trees as burn-in.

A Bayesian maximum clade credibility tree of 2170 posterior distribution trees showed a strongly supported divergence for the *B. stacei* and *B. distachyon* lineages that split from the common ancestor 11.5 Ma (Fig. 4a; Supplementary Fig. 7). Noticeably, the divergence time of the D subgenomes of the *B. hybridum-*S-plastotype clade from its parental *B. distachyon* lineage (0.14 Ma) is less than the divergence between some of the *B. distachyon* groups (0.16 Ma; Fig. 4a; Supplementary Fig. 7). While the divergence times for the *B. distachyon* groups are confounded by introgression between groups, this only makes the recent divergence of the D subgenomes of the *B. hybridum-*S-plastotype clade more remarkable. The density tree cladogram showed a high congruence of most posterior distribution trees with this topology (Supplementary Fig. 8).

The cross-bracing prior approach of McCann et al.^30^ was adapted to SNAPP in order to obtain a single-age estimate for the respective origins of the ancestral (D plastotypes) and the recent (S plastotypes) clades of the *B. hybridum* allotetraploids. It was justified by the fact that the splits corresponding to the same hybridization events had nearly the same age (Fig. 4a). We imposed a cross-bracing normal distribution prior with mean 0 and a standard deviation of 0.02, enforcing very low probability on trees that differ in node height and making the age distributions of the cross-braced nodes become nearly congruent with respect to mean and shape^30^. Prior distributions were generated for the split times between *B. stacei* and *B. hybridum*-S-subgenomes and for *B. distachyon* and *B. hybridum*-D-subgenomes and their arithmetic difference in two separate searches, one for the age of the ancestral *B. hybridum* D plastotypes and other for those of the recent *B. hybridum-*S plastotypes. We implemented the cross-bracing approach in SNAPP by manually adding several blocks containing parameter, prior, and operator commands in the Beauty xml file.

### K-mer analysis

Transposable elements, TEs, turnover rapidly in genomes and can proliferate over short timescales making them useful for detecting the divergence between closely related genomes^74^. Detecting different k-mer conservation rules between different strains can serve as a proxy to reconstruct the history of repetitive sequences (transposons and other types of repeats) among the lines and to elucidate differences in their recent evolutionary history. K-mers of length 26 were counted for eight *B. hybridum* lines, eight *B. distachyon* lines, and one *B. stacei* line (Supplementary Data 4) using polyCRACKER^75^. Representative *B. distachyon* lines were selected based on dimensionality reduction and clustering of a k-mer minhash distance matrix found via sourmash (v2.0.0)^76^. The most representative members of these clusters, closest to the centroids, were selected for the analysis along with the reference *B. distachyon* Bd21 genome.

The k-mers found after this clustering step were considered to be part of a particular k-mer conservation class, and were visualized and analyzed using an interactive Plotly Dash (v0.30.0) application. The relative distribution of k-mers across the studied lines of each analysis was shown on boxplots with their box points and a descriptive PCA plot detailing k-mer abundance for particular rules. The post-assembled distributions of k-mers were compared with their respective pre-assembled distributions, corroborating that their observed conservation patterns did not correspond to a failure to assemble repeats in certain assemblies or due to scaffolding artifacts. The retrieved conservation classes of the k-mers highlighted their abundance or sparsity along the different studied lines and subgenomes, illustrating the origins of these k-mers from the proliferation of other repetitive sequences.

While variation in the completeness of the genome assemblies can impact this analysis, we do not believe this affected our main conclusions for three reasons. First, 13 of the 17 lines analyzed were similar quality Illumina assemblies. Second, the higher-quality assemblies always grouped together consistently with the most closely related lines. For example, all six *B. hybridum-*S-plastotype lines show very similar patterns, despite the fact that Bd28 was a PacBio assembly, ABR113 was a high-quality reference assembly and the other four lines were Illumina assemblies. Third, the subgenomes in each *B. hybridum* line had very different k-mer patterns. That said, the relative abundance of k-mers in classes six and seven may be affected by assembly quality since the lines with the highest abundance of those k-mers, Bd21, Bd28, ABR113 are also the lines with the best assemblies.

### Pan-genome analysis

CDS sequences from primary transcripts annotated in four of the newly assembled *B. hybridum* genomes (ABR113 reference genome, Bhyb26 D plastotype, and Bd28 and Bhyb30 S plastotypes; Supplementary Data 4) were assigned to S and D subgenomes. The resulting FASTA files were analyzed with GET_HOMOLOGUES-EST v09052018^77^ together with CDS sequences from *B. stacei* (ABR114) and 52 *B. distachyon* lines. Nucleotide-based sequence clusters were produced with the MCL algorithm (-M) requiring alignment coverage of at least 75% (-C 75) and no sequence identity restriction (-S 1). The resulting clusters were then sorted, and a pan-genome matrix was produced summarizing the occupancy of each gene across the whole set of genomes and subgenomes. Finally, the pan-genome matrix was interrogated in order to obtain the number of core, soft-core and shell genes. Detailed protocols and sequence files are available at http://floresta.eead.csic.es/plant-pan-genomes/Bhybridum.

While assembly quality could affect the pan-genome analysis, we do not think this caused significant bias because low complexity sequences like genes are efficiently assembled in the compact *Brachypodium* genomes. In addition, the fact that the three higher-quality assemblies appear indistinguishable from the other assemblies indicates that assembly quality is not a systematic source of error.

### Selection analysis

Selection rates were estimated using dN/dS nucleotide substitution rate ratio in the D and S genic groups that contained 7228 and 9432 orthologs (CDSs), respectively (Supplementary Data 4). The smaller S group (one *B. stacei* ABR114 genome and five *B. hybridum* Bhyb26, Bhyb118-5, ABR113, Bd28, Bhyb30 S subgenomes) had more genes orthologous to the *B. distachyon* Bd21 outgroup genome than the large D group (44 *B. distachyon* genomes and 5 *B. hybridum* D subgenomes) to the *B. stacei* ABR114 outgroup genome. Pairwise dN/dS values were calculated for all samples and genes within each group using their respective outgroup sequence (Supplementary Data 11). Basic statistics (mean, median, SD, range) of the dN/dS values were computed for each sample, and the total number of genes using the Rstat package v. 4.1.0 (R core team) and their distributions were plotted using box and whisker plots. Considering the nonnegative distribution of dN/dS values, statistical significant differences between median dN/dS values of samples within each D and S group were tested using a GLM test with a gamma distribution, which tests for significant differences, a Wilcoxon pairwise difference test for all pairs of samples, and a Kruskal–Wallis rank test for the whole group of samples within each group using the respective options of the Rstat package. Further statistical analyses were conducted using the lsmeans package of R to compute posthoc Tukey tests for the least square means.

### Reference-based analysis

Illumina reads for each line (except for Bd28 for which simulated Illumina short-reads were created from the final PacBio genome assembly) were aligned to Bd21-3 v1.1 reference genome with BWA (v0.7.17)^78^, filtered with Picard tools (v2.18) FixMateInformation and MarkDuplicates (https://broadinstitute.github.io/picard), then GATK (v4.0)^79^ was used for base-quality score recalibration, indel realignment, and SNV and InDel discovery using standard hard filtering parameters from GATK Best Practices recommendations. Areas with a read depth <3 were counted as deletions or highly divergent regions.

### Gene expression analysis

RNA-seq data were obtained for *B. hybridum* ABR113 leaves and spikelets. Leaf samples were from seedlings at the 3–4 leaf stage. Each spikelet sample consisted of the spikelets from one plant, with each spikelet collected separately 3 days after inflorescence emergence. RNA was extracted using TRIzol (Life Technologies) or PureLink (ThermoFisher) kits, DNAse-treated using the DNA-free™ Kit (Life Technologies), and RNA quality was assessed by Nanodrop (ThermoFisher), agarose gel, and BioAnalyzer (Agilent). Strand-specific libraries were prepared using the Illumina TruSeq kit, and library quality was checked by BioAnalyzer (Agilent). Libraries were sequenced using Illumina technology. The average number of total mapped paired-end reads ranged from ~60 to ~100 million reads.

Raw RNA-seq reads were filtered and trimmed using BBDuk (v37) from the BBtools package (v. 38.0)^80^. Reads were aligned to the complete reference genome (ABR113 v. 1.0) using BBmap (v37)^80^. To increase mapping stringency, reads were required to share 90% sequence identity with the target location, and ambiguous reads were discarded. With these criteria, more than 96% of reads were mapped unambiguously. There were ~194 genes, which were associated with five or more ambiguous reads. Gene-level counts were obtained using HTSeq (v. 0.9.1)^80^. Transcripts per million (TPM) values were calculated using a custom Python script.

To test for homeolog-expression bias using DESeq2 v. 1.24.0^81^, each library was split into two, with each subgenome of each library becoming one sample. Homeologs were determined using Phytozome’s Phytomine pipeline, incorporating both homology and synteny^82^. Genes lacking a 1:1 homeolog were excluded from the DESeq2 analysis. The length of each homeolog was added to the DESeqDataSet using avgTxLength. This lengths matrix was incorporated into normalization factor estimation, which is in turn used to estimate each gene’s true expression value. The normalization factor option was designed mainly to account for differences in alternative transcript usage between conditions, but can be used to control for any known systematic bias for a given gene between two samples. Our models for the likelihood ratio test (using Wilkinson notation) were: full=library+subgenome, and reduced=library, so library was essentially handled as a batch effect.

Our candidate pseudogene identification protocol was based on the method of Session^83^. *Brachypodium hybridum* subgenomes were compared with their cognate diploid genomes three genes at a time to identify triplets where the central gene lacked a *B. hybridum* ortholog but the two flanking genes each had a syntenic *B. hybridum* ortholog. The *B. hybridum* genomic region between the two flanking orthologs was extracted using bedtools (v2.27.1)^84^, and the diploid gene was aligned to this sequence using the codon- and intron-aware protein2genome model of exonerate (v. 2.4.0) (https://www.ebi.ac.uk/about/vertebrate-genomics/software/exonerate). Alignments were parsed with the Biopython (v. 1.7.0) package ExonerateIO. RNA-seq counts were obtained strictly for the aligned regions using the customizable functionalities of HTSeq, and normalized by the total number of mapped reads for each library. Pairwise nonsynonymous to synonymous substitution rate ratios were calculated using the yn00 program from PAML (v. 4.9 h)^85^. Finally, candidate pseudogenes were required to have a nonsynonymous to synonymous substitution rate ratio (dN/dS) of 0.5 or higher, and the log ratio of diploid to polyploid expression for the aligned region had to be >0.3.

### Reporting summary

Further information on research design is available in the Nature Research Life Sciences Reporting Summary linked to this article.

## Supplementary information

Supplementary Information

Peer Review File

Reporting Summary

Description of Additional Supplementary Files

Supplementary Data 1

Supplementary Data 2

Supplementary Data 3

Supplementary Data 4

Supplementary Data 5

Supplementary Data 6

Supplementary Data 7

Supplementary Data 8

Supplementary Data 9

Supplementary Data 10

Supplementary Data 11

## Data Availability

Data supporting the findings of this work are available within the paper and its Supplementary Information files. A reporting summary for this Article is available as a Supplementary Information file. The datasets generated and analyzed during the current study are available from the corresponding author upon request. Genome assemblies and annotations can be downloaded from Phytozome [https://phytozome.jgi.doe.gov/]. The direct link for the *B. hybridum* ABR113 reference genome is https://phytozome-next.jgi.doe.gov/info/Bhybridum_v1_1; the direct link for the *B. stacei* ABR114 reference genome is https://phytozome-next.jgi.doe.gov/info/Bstacei_v1_1; and the direct link for the *B. distachyon* Bd21 reference genome is https://phytozome-next.jgi.doe.gov/info/Bdistachyon_v3_1. The other genome assemblies and annotations created in this study (listed in Supplementary Data 1) can be downloaded from the *B. hybridum* genome page [https://phytozome-next.jgi.doe.gov/info/Bhybridum_v1_1] through the download directory labeled “Additional genomes used in Gordon et al. *Nat. Commun.* 2020” direct link https://genome.jgi.doe.gov/portal/pages/dynamicOrganismDownload.jsf?organism=Bhybridum. Note that a free account is required to download the data from Phytozome. The raw reads for the genomic sequences and RNA sequences are available from NCBI or ENA. The samples numbers, accessions, and hyperlinks are provided in Supplementary Data 4 and 9. Seeds for the lines used in this study are available from the USDA National Plant Germplasm Service or by request from the authors.The source data underlying Figs. 6, 7, and 8 and Supplementary Figs. 9, 11, and 12 are provided as a Source Data file.

## Source data

Source Data
