# Supplementary material for: Gradual polyploid genome evolution revealed by pan-genomic analysis of Brachypodium hybridum and its diploid progenitors
Source: Nat Commun. 2020 Jul 29;11:3670. doi: 10.1038/s41467-020-17302-5 (PMC7391716; doi:10.1038/s41467-020-17302-5)
Supplement: Supplementary file 1 — Supplementary Information [file 41467_2020_17302_MOESM1_ESM.pdf]

Gradual polyploid genome evolution revealed by pan-genomic analysis of *Brachypodium hybridum* and its diploid progenitors

Gordon *et. al.*

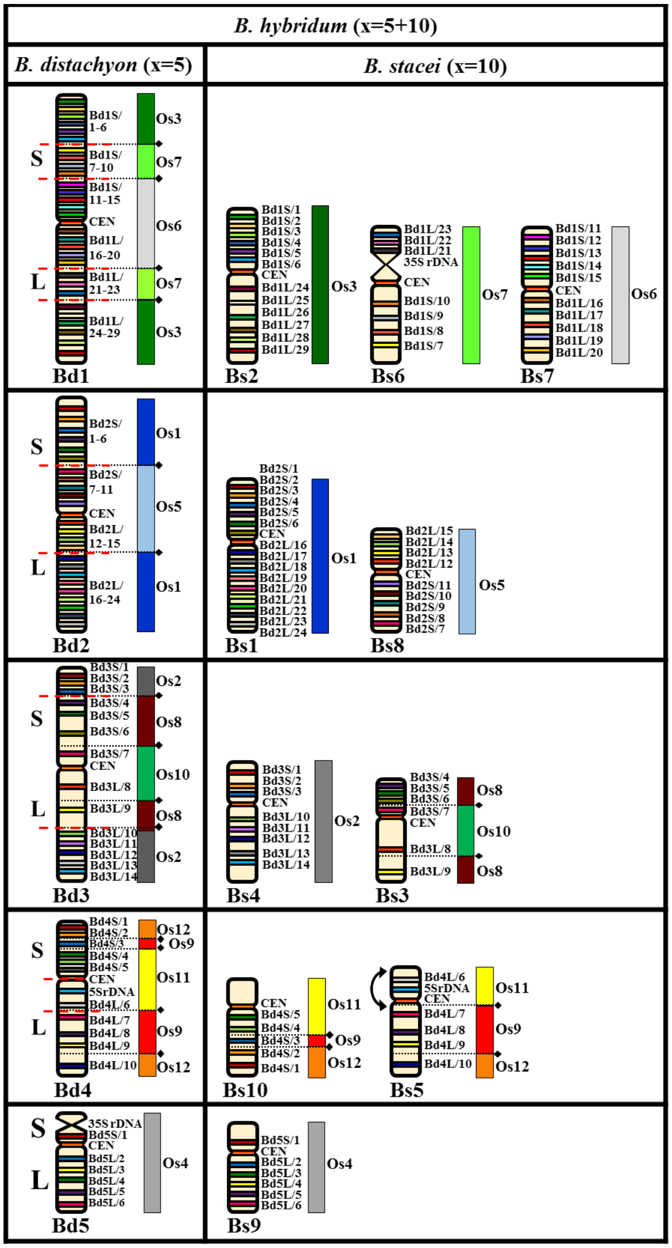

Supplementary Figure 1. Chromosomal distribution of the single-locus BAC clones that were derived from chromosomes Bd1-Bd5 of *B. distachyon* and mapped using fluorescence *in situ* hybridization-based comparative chromosome barcoding to the chromosomes of *B. stacei* and *B. hybridum*. The distribution of the clones on the chromosome diagram reflects their position on the physical map of the *B. distachyon* genome. Detailed lists of BAC clones used in the comparative cytomolecular analyses are shown in Supplementary table 1. The diagrams next to the *Brachypodium* chromosomes relate the BAC clones to the homeologous regions in different rice (Os) chromosome equivalents (intermediate ancestral grass chromosomes). Black diamonds and dotted lines indicate the hypothetical fusion points of the intermediate ancestral grass chromosomes in Bd1-Bd5. Red dashed lines indicate the chromosomal breakpoints that were found in the Bs-genome chromosomes in *B. stacei* and *B. hybridum*. Arrows point to the inversion that was present on chromosome Bs5 of these species.

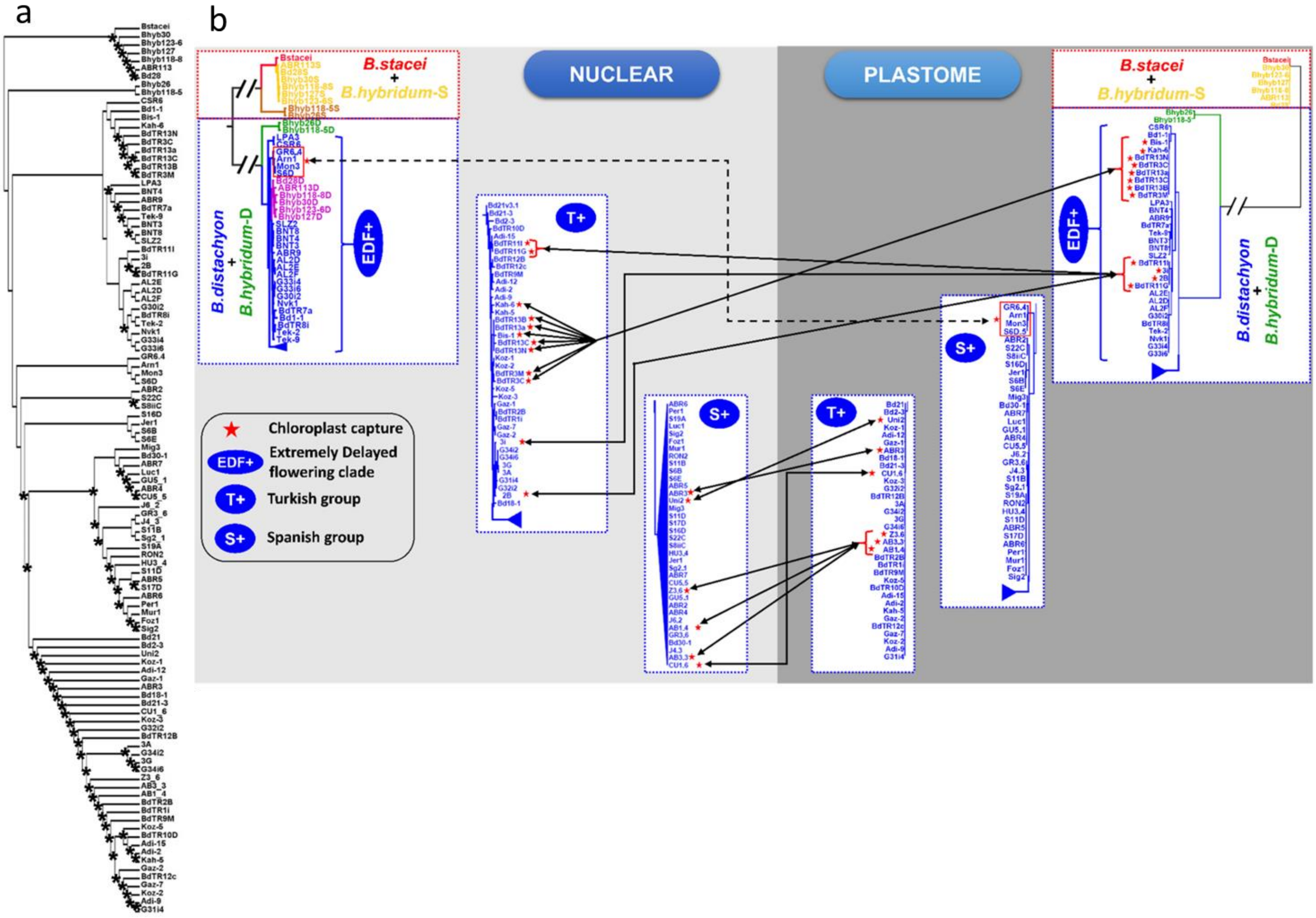

Supplementary Figure 2. Chloroplast capture. a) Cladogram of Maximum Likelihood *Brachypodium* phylogenomic tree based on plastomes constructed with IQTREE showing high ultrafast bootstrap branch support of main clades and groups (asterisks indicate branches with support < 95%). b) Comparative evolutionary nuclear and plastid analysis. Nuclear (left) vs plastome (right) trees. Stars and arrows indicate chloroplast capture events between the main *B. distachyon* groups. *B. stacei (red), B. hybridum* S-plastotypes plastomes and nuclear S subgenome (yellow), *B. hybridum* S-plastotypes nuclear D subgenome (purple), *B. hybridum* D-plastotypes plastomes and nuclear D subgenome (green), *B. hybridum* D-plastotypes nuclear S subgenome (brown), *B. distachyon* (blue).

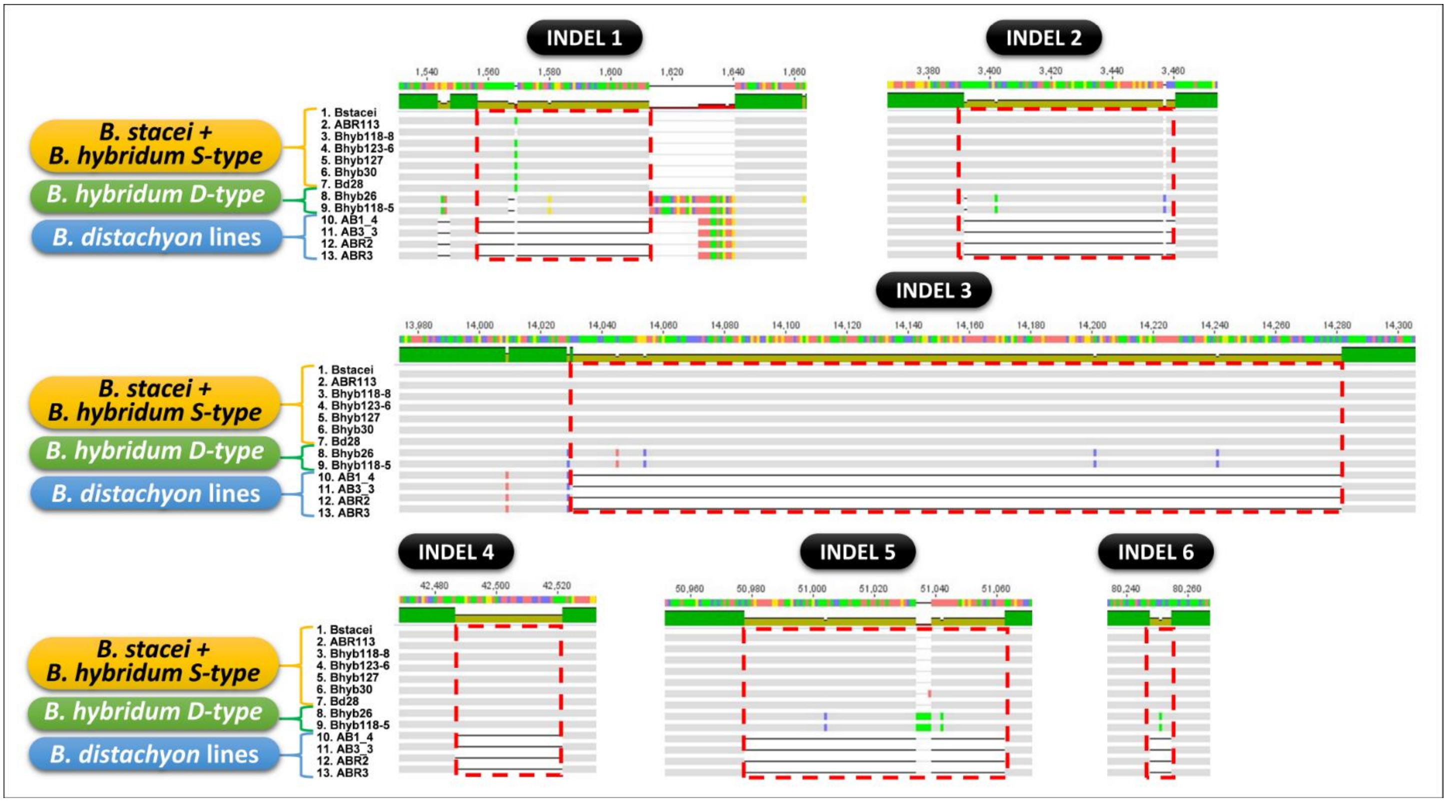

Supplementary Figure 3. Plastome insertions shared by *B. stacei* and all studied *B. hybridum* plastomes (D and S plastotypes). Indel 1: 56-68 bp (start position 1,557); Indel 2: 68 bp (3,392); Indel 3: 251 bp (14,031); Indel 4: 35 bp (42,487); Indel 5: 80-85 bp (50,978); Indel 6: 7 bp (80,248).

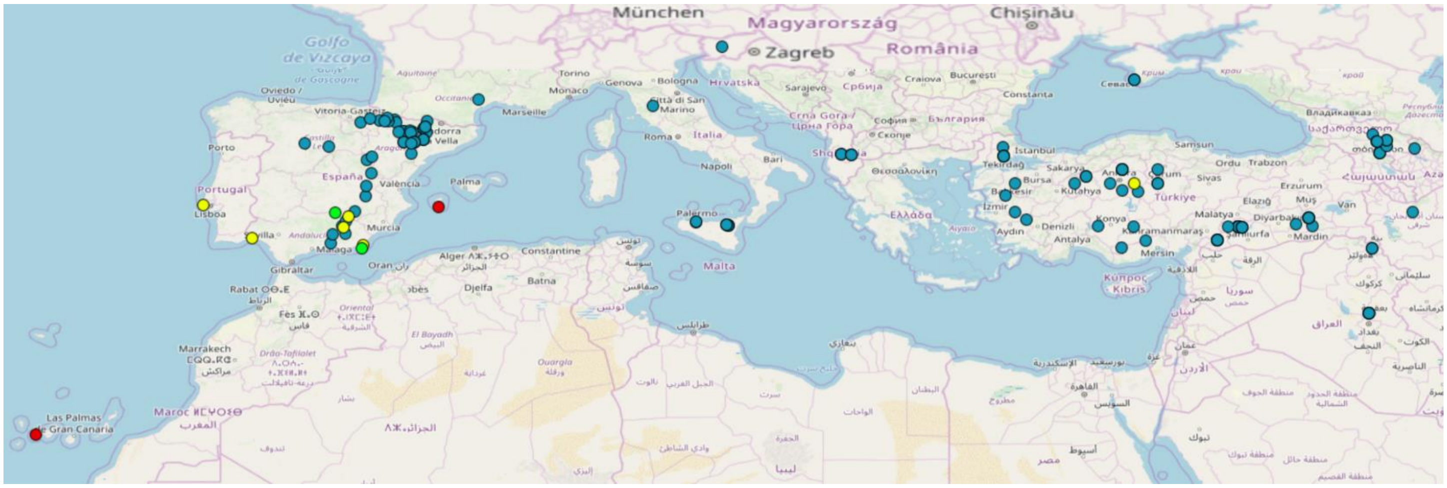

Supplementary Figure 4. Geographical origin of the lines used in this study. *B. distachyon* (120 lines, blue circles), *B. stacei* (2 lines, red circles), *B. hybridum* D-plastotype (2 lines, green circles), *B. hybridum* S-plastotype (6 lines, yellow circles). See Supplementary data 4 for coordinates used to make this figure. The invasive Australian *B. hybridum* B28 line (S-plastotype) is not shown in this map. Note that some lines overlap because they have the same or very close geographical coordinates.

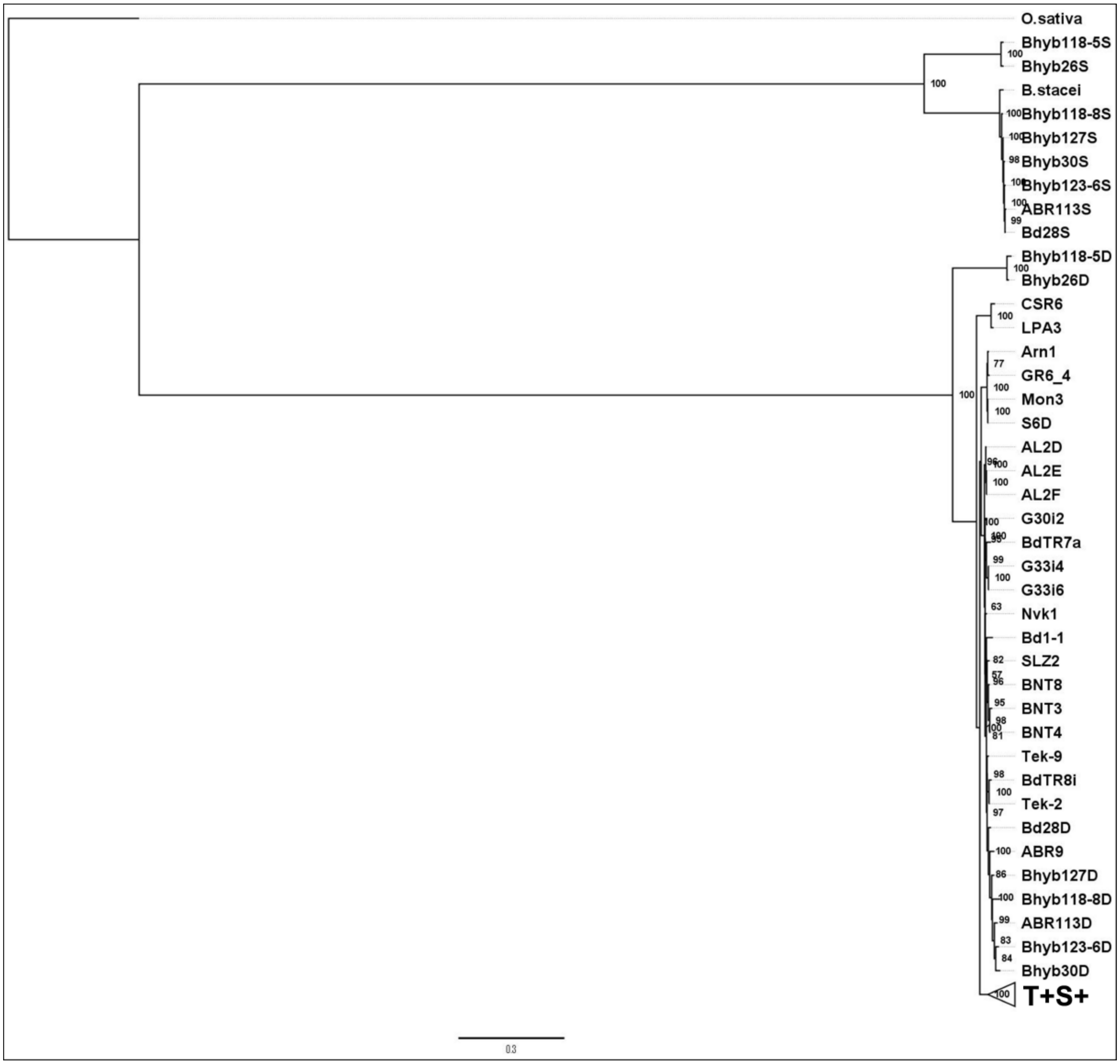

Supplementary Figure 5. Maximum likelihood phylogenomic tree of 116 *B. hybridum, B. distachyon* and *B. stacei* lines based on a reduced data matrix of 5,443 syntenically aligned nuclear SNVs that were also homologous to the 12 chromosomes of the outgroup *Oryza sativa.*

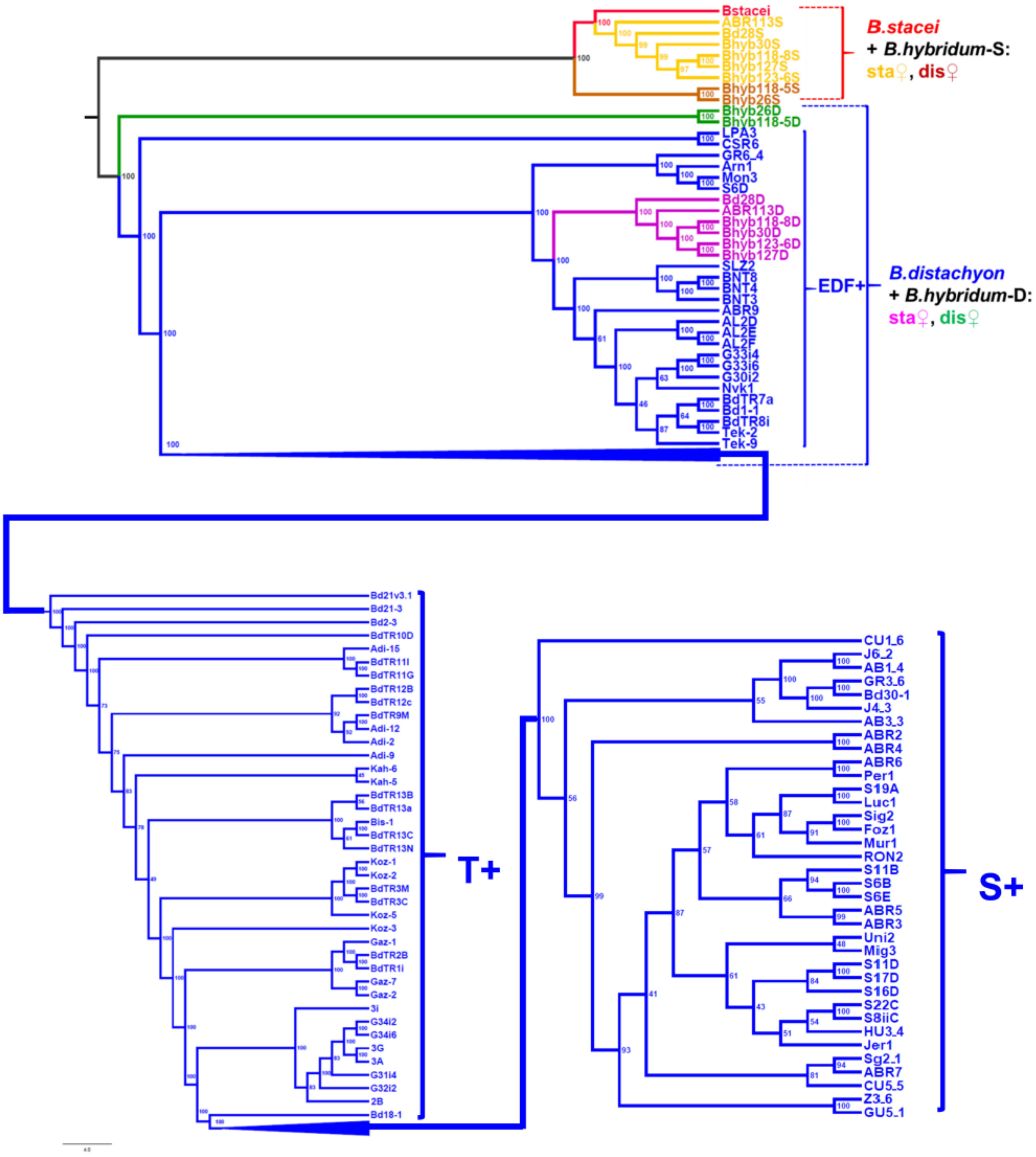

Supplementary Figure 6. Best Maximum Likelihood *Brachypodium* phylogenomic tree based on 745,858 syntenically aligned nuclear SNPs constructed with IQTREE. Cladogram showing branch support. *B. stacei* (red), *B. distachyon* (blue), *B. hybridum* D plastotypes S subgenome, brown; D subgenome, green), *B. hybridum* S plastotype (S subgenome, yellow; D subgenome, purple). This is the same tree as Fig. 4a, but all lines are legible and bootstrap support is indicated.

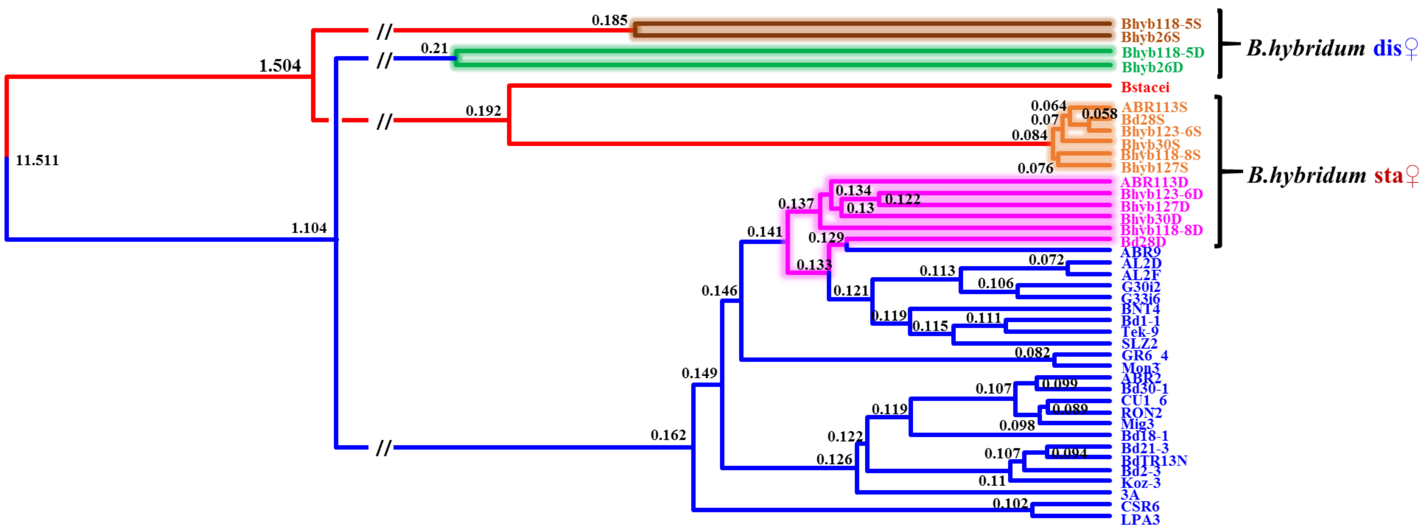

Supplementary Figure 7. Dated *Brachypodium* maximum clade credibility tree based on 4,942 nuclear SNVs constructed using the multi-species coalescence model with SNAPP. Estimated split times (million years ago) for the crown nodes and the successive diverging nodes of the *B. stacei* (red) and *B. distachyon* (blue) groups are indicated on branches. Subgenomic lineages of ancestral *B. hybridum* D plastotypes are shown in green (D subgenome) and brown (S subgenome) and those of the recent *B. hybridum* S plastotypes in purple (D subgenome) and orange (S subgenome).

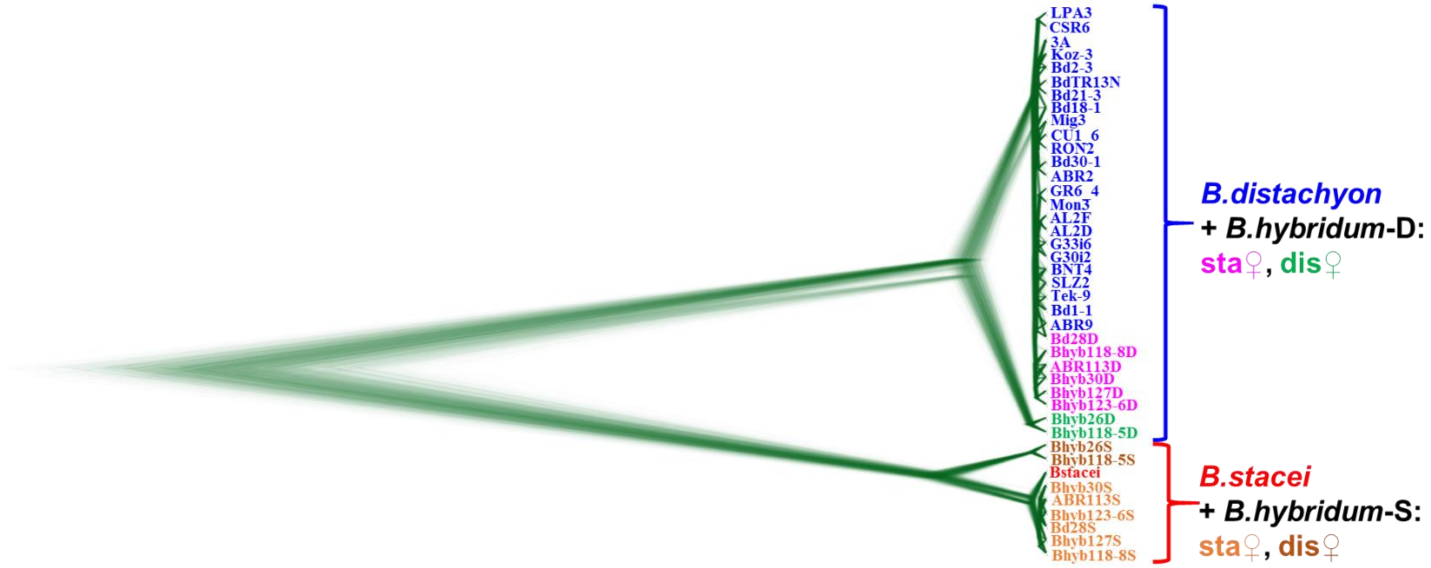

Supplementary Figure 8. Bayesian *Brachypodium* phylogenetic trees based on 4,942 nuclear SNVs constructed using the multi-species coalescence model with SNAPP. Density tree of 2,170 trees. *B. stacei* (red); *B. distachyon* (blue); *B. hybridum* D plastotypes: D subgenome (green), S subgenome (brown); *B. hybridum* S plastotypes: D subgenome (purple), S subgenome (orange).

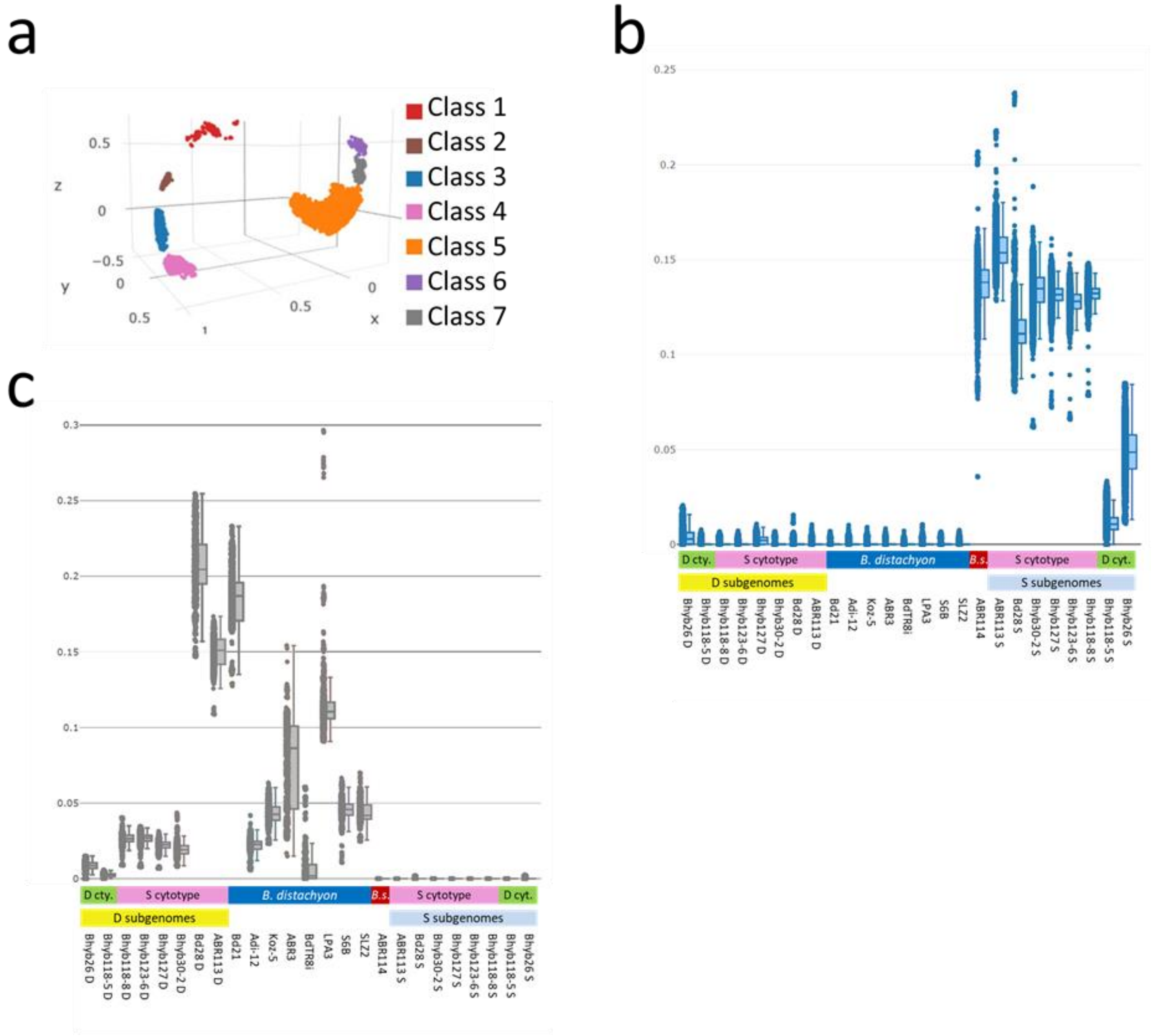

Supplementary Figure 9. K-mer abundance graphs for the classes not shown in Fig. 6. (a) Three-dimensional kernel PCA (cosine kernel) plot showing the seven distinct classes of k-mers identified by the algorithm. Class 3 (b) and Class 7 (c) on each genome are plotted for each k-mer class designated by color (see Fig. 6). The species/subgenome and plastotype are indicated below the x-axis; accession codes correspond to those indicated in Supplementary data 4. B.s.=*B. stacei*. Dots represent relative abundance of individual k-mers. Boxplots show the median and 25-75% range. Error bars are +/- 1 sd. Source data are provided as a Source Data file.

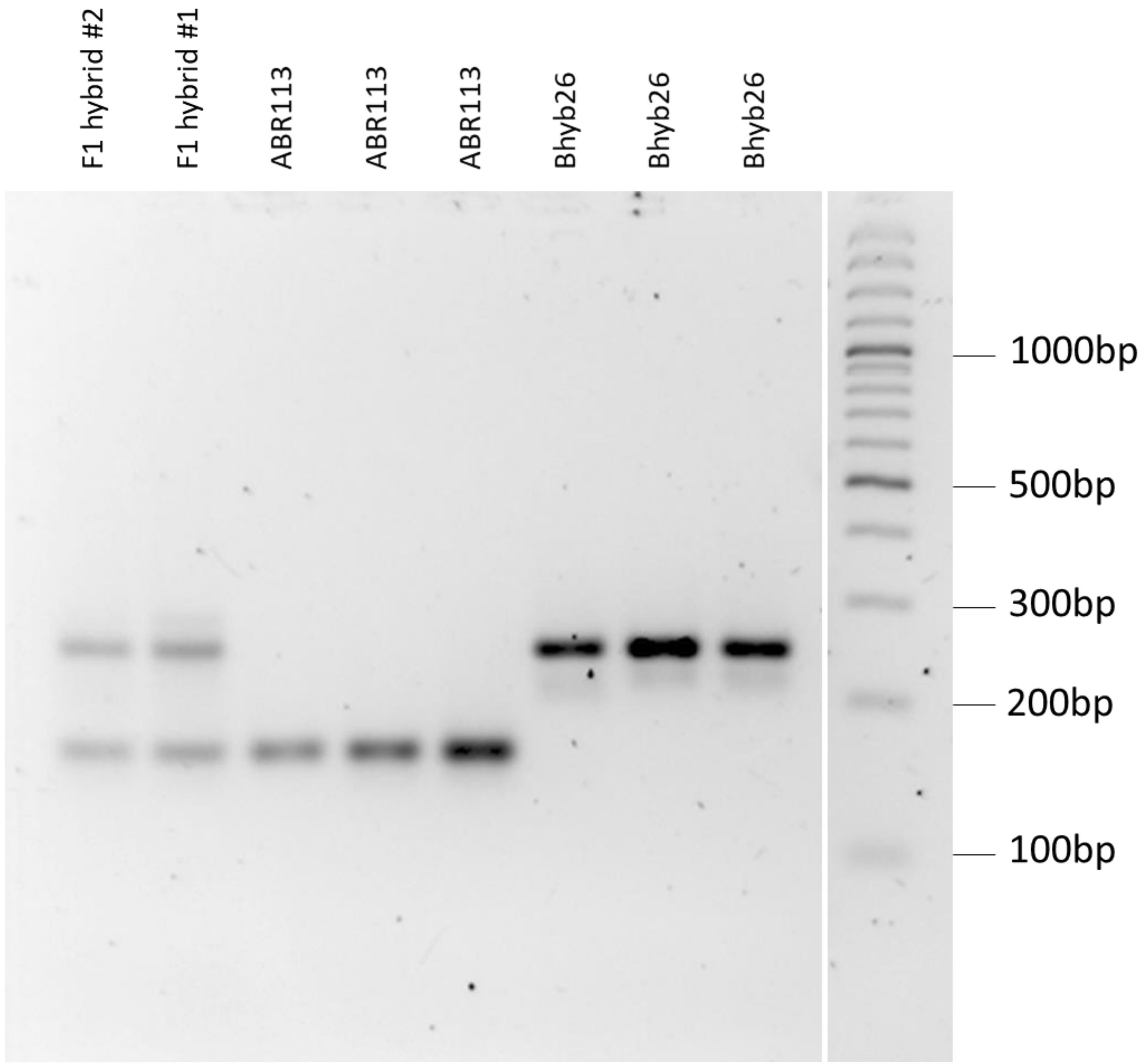

Supplementary Figure 10. PCR verification of F1 hybrids. Example of a genotyping gel for progeny of ABR113 x Bhyb26 crosses. A representative gel from one of 3 primer pairs from different locations throughout the genome, which reliably and unambiguously distinguished the *B. hybridum* ABR113 and Bhyb26 accessions, is shown. Note that the F1 hybrids contains both parental band sizes. Primer pair shown: 5-’CCTATGGCAATTTTGGAGACG-3’, 5’-CCTATTCTTCTGATCCAAGAGATCC-3’. Additional informative primer pairs: 5’-GAGTCCGGATAAGTGCAGCCC-3’ and 5’-CTTGGGCAAACCACGACTGCT-3’; 5’-GTCTGGTCGTGCTCGCAATACGC-3’ and 5’-GCGATGCATGTCATGTGAGTG-3’.

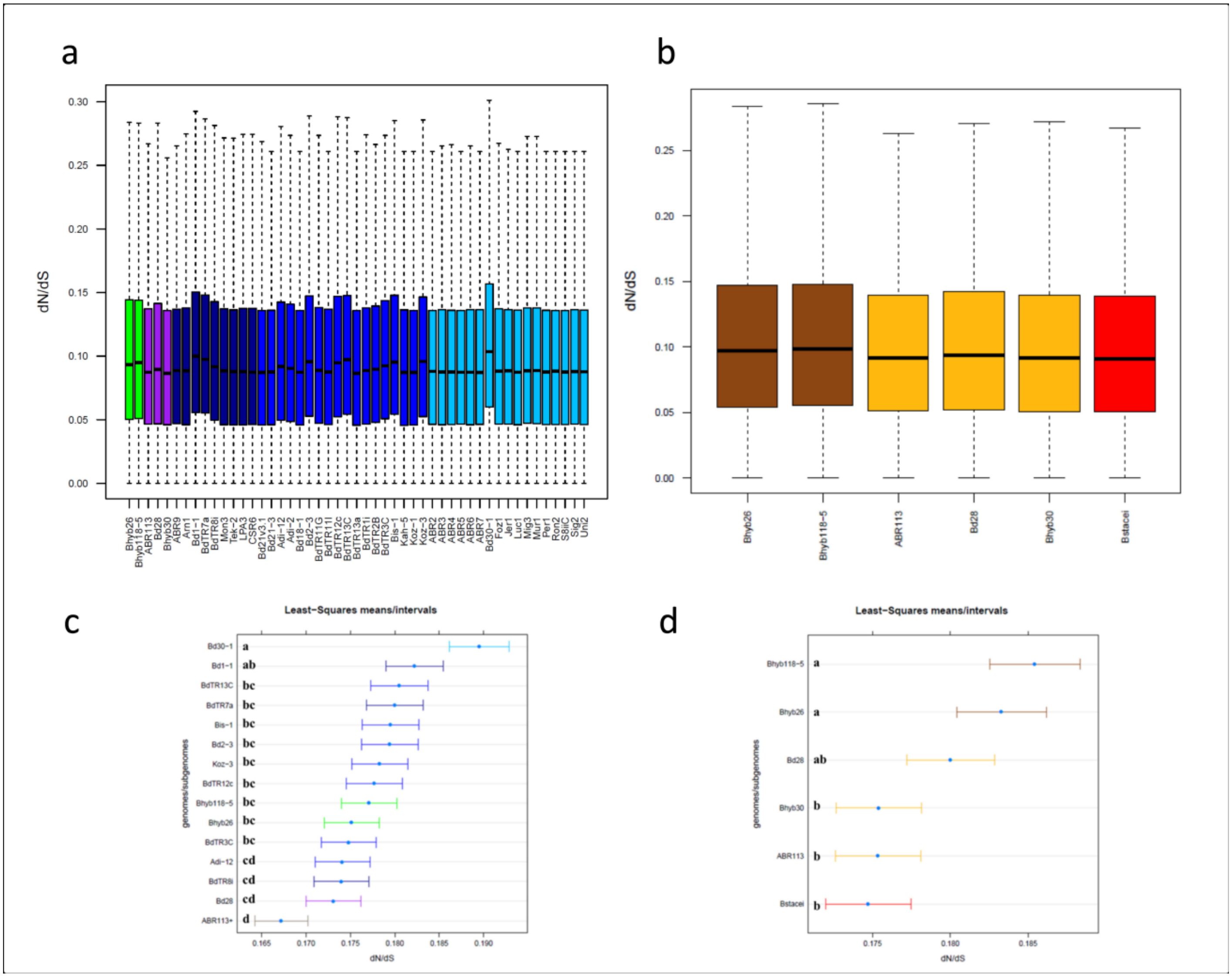

Supplementary Figure 11. Selection analysis. Distribution of dN/dS values of genes under negative selection pressure (dN/dS<0.5) in *B. distachyon* genomes and *B. hybridum* D subgenomes (a) and in *B. stacei* genome and *B. hybridum* S subgenomes (b) groups. Box plots depict the corresponding median, the first (25%) and the third (75%) quartiles and whisker plots depict 1.5 times the interquartile range. (c and d) Least-Squares means and confidence intervals (P<0.05) obtained through the Generalized Lineal Model (GLM) of the R program for, respectively, the (a) and (b) groups. Equal letters indicate no significant differences among genomes. Note the less constrained dN/dS values for the two *B. hybridum* D plastotypes Bhyb26 and Bhyb118-5 and the S plastotype Bd28 genes as well as for 12 *B. distachyon* lines genes with respect to the rest. Source data are provided as a Source Data file.

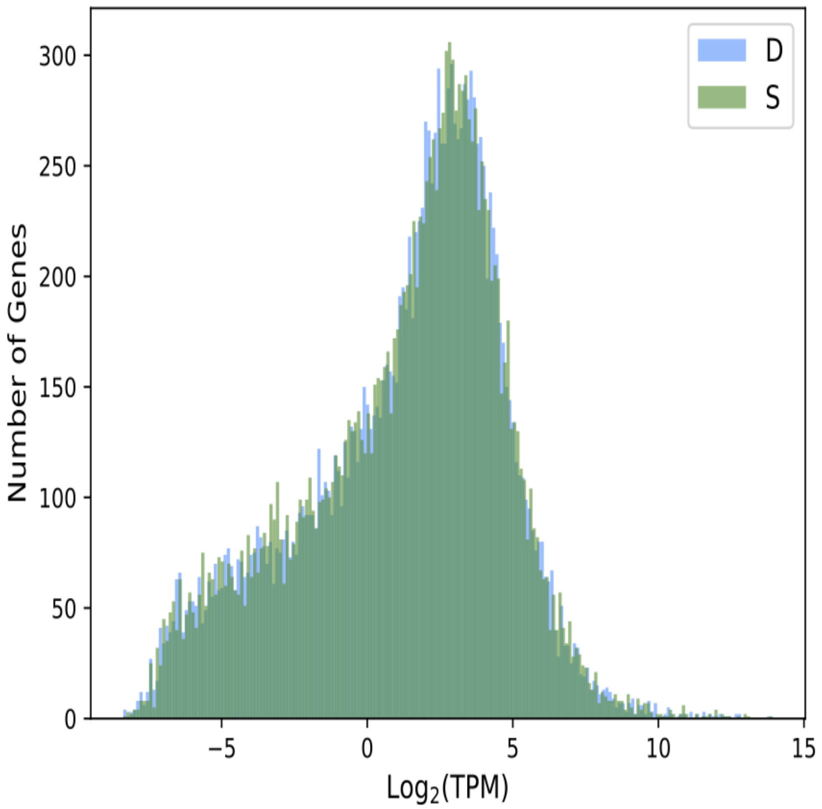

Supplementary Figure 12. Expression analysis. Normalized expression values for homeologous genes of the *B. hybridum* ABR113 subgenomes D (blue) and S (green) obtained by mapping to the entire reference genome. The similar distributions demonstrate a lack of obvious preferential subgenome expression. Non-expressed genes were included in statistical tests but removed from graph for visual clarity. Source data are provided as a Source Data file.

Supplementary Table 1. BAC clones used in the comparative cytomolecular analyses.

BAC name	BAC clone identifier*	Position in the genome (bp)
CEN	BD_CBa0033J12	-
Bd1S/1	BD_CBa0027N17	Bd1: 560624 : 710332
Bd1S/2	BD_ABa0004B12	Bd1: 3276891 : 3460444
Bd1S/3	BD_ABa0017K22	Bd1: 5375697 : 5509098
Bd1S/4	BD_CBa0030L10	Bd1: 8680898 : 8845282
Bd1S/5	BD_ABa0032D10	Bd1: 9006125 : 9148678
Bd1S/6	BD_ABa0027D04	Bd1: 12706461 : 12847057
Bd1S/7	BD_ABa0020A04	Bd1: 15092918 : 15238493
Bd1S/8	BD_ABa0009N18	Bd1: 17150298 : 17335777
Bd1S/9	BD_CBa0002O16	Bd1: 20013520 : 20160236
Bd1S/10	BD_CBa0022H13	Bd1: 23114454 : 23242441
Bd1S/11	BD_ABa0018O15	Bd1: 25727688 : 25878318
Bd1S/12	BD_ABa0044I06	Bd1: 28135872 : 28292480
Bd1S/13	BD_ABa0036J15	Bd1: 31222238 : 31387974
Bd1S/14	BD_CBa0024I19	Bd1: 32507293 : 32633286
Bd1S/15	BD_ABa0004L01	Bd1: 34316249 : 34466638
Bd1L/16	BD_ABa0002I22	Bd1: 39952805 : 39424325
Bd1L/17	BD_CBa0004P09	Bd1: 43536825 : 43670757
Bd1L/18	BD_CBa0025P22	Bd1: 46564769 : 46692954
Bd1L/19	BD_CBa0044L08	Bd1: 48612347 : 48783561
Bd1L/20	BD_ABa0003G14	Bd1: 50139085 : 50274374
Bd1L/21	BD_CBa0035K24	Bd1: 51720482 : 51914140
Bd1L/22	BD_ABa0046G17	Bd1: 54082210 : 54253048
Bd1L/23	BD_CBa0028P17	Bd1: 57093738 : 57225377
Bd1L/24	BD_ABa0010A14	Bd1: 59503419 : 59676696
Bd1L/25	BD_ABa0013D23	Bd1: 64120769 : 64297730
Bd1L/26	BD_ABa0043A05	Bd1: 68898017 : 69053532
Bd2S/1	BD_ABa0038A01	Bd2: 1022 : 132144
Bd2S/2	BD_ABa0027K15	Bd2: 1864643 : 2004976
Bd2S/3	BD_ABa0028O04	Bd2: 3492740 : 3587755
Bd2S/4	BD_ABa0045F24	Bd2: 6004397 : 6146555
Bd2S/5	BD_ABa0012B07	Bd2: 9006125 : 9148678
Bd2S/6	BD_ABa0005E09	Bd2: 10380990 : 10507985
Bd2S/7	BD_CBa0023P23	Bd2: 10380990 : 10507985
Bd2S/8	BD_ABa0017D02	Bd2: 15866689 : 16021967
Bd2S/9	BD_ABa0047D12	Bd2: 17856422 : 17996794
Bd2S/10	BD_ABa0026K14	Bd2: 19861012 : 20005795
Bd2S/11	BD_CBa0038L02	Bd2: 22509927 : 22639901
Bd2L/12	BD_ABa0014K11	Bd2: 34309867 : 34503922
Bd2L/13	BD_CBa0007E06	Bd2: 36376507 : 36505573
Bd2L/14	BD_CBa0022I07	Bd2: 38509106 : 38646001
Bd2L/15	BD_CBa0016E24	Bd2: 39997753 : 40003453
Bd2L/16	BD_ABa0008H07	Bd2: 42500887 : 42664133

BAC name	BAC clone identifier*	Position in the genome (bp)
Bd2L/17	BD_CBa0041G17	Bd2: 44876290 : 45007631
Bd2L/18	BD_CBa0031I09	Bd2: 46500135 : 46639653
Bd2L/19	BD_CBa0019P09	Bd2: 48369110 : 48504229
Bd2L/20	BD_CBa0038L04	Bd2: 50005019 : 50143082
Bd2L/21	BD_ABa0031O24	Bd2: 52001822 : 52162247
Bd2L/22	BD_CBa0036G07	Bd2: 53816466 : 54010118
Bd2L/23	BD_CBa0047O03	Bd2: 55698147 : 56502216
Bd2L/24	BD_ABa0038G14	Bd2: 57002804 : 57148130
Bd3S/1	BD_CBa0028O16	Bd3: 856255 : 1007650
Bd3S/2	BD_ABa0015A18	Bd3: 4001904 : 4157452
Bd3S/3	BD_ABa0018B12	Bd3: 6003300 : 6153924
Bd3S/4	BD_ABa0030J22	Bd3: 8504730 : 8651070
Bd3S/5	BD_CBa0016A22	Bd3: 11505050 : 11712720
Bd3S/6	BD_ABa0022G01	Bd3: 16038657 : 16055486
Bd3S/7	BD_ABa0033D16	Bd3: 22106200 : 22299788
Bd3L/8	BD_CBa0011M04	Bd3: 36854229 : 37002472
Bd3L/9	BD_ABa0038N13	Bd3: 44001051 : 44142538
Bd3L/10	BD_ABa0026M18	Bd3: 49347850 : 49503810
Bd3L/11	BD_ABa0037F15	Bd3: 50854746 : 51004145
Bd3L/12	BD_ABa0037C10	Bd3: 52501229 : 52687354
Bd3L/13	BD_ABa0008G22	Bd3: 55503533 : 55665230
Bd3L/14	BD_ABa0020N10	Bd3: 57504387 : 57653389
Bd4S/1	BD_CBa0030B12	Bd4: 2007584 : 2157984
Bd4S/2	BD_CBa0040J03	Bd4: 7830905 : 8001843
Bd4S/3	BD_CBa0021B09	Bd4: 9502901 : 9667864
Bd4S/4	BD_ABa0043D11	Bd4: 11006774 : 11150531
Bd4S/5	BD_ABa0010I18	Bd4: 14002249 : 14164264
Bd4L/6	BD_ABa0006J17	Bd4: 29358544 : 29516826
Bd4L/7	BD_ABa0020D08	Bd4: 32504625 : 32642850
Bd4L/8	BD_CBa0035E05	Bd4: 39350118 : 39526113
Bd4L/9	BD_CBa0038H23	Bd4: 42789149 : 43003220
Bd4L/10	BD_ABa0041I03	Bd4: 48350055 : 48507632
Bd5S/1	BD_ABa0019O20	Bd5: 1091367 : 1236179
Bd5L/2	BD_ABa0045F23	Bd5: 13499779 : 13653343
Bd5L/3	BD_ABa0023L21	Bd5: 17634500 : 17679830
Bd5L/4	BD_CBa0024J19	Bd5: 20845837 : 21003148
Bd5L/5	BD_CBa0032J06	Bd5: 23870997 : 24003288
Bd5L/6	BD_ABa0019J13	Bd5: 25906054: 26098440

* More detail can be found in the NCBI database under the following URLs:

http://www.ncbi.nlm.nih.gov/clone/library/genomic/424 (BD_ABa library) and

http://www.ncbi.nlm.nih.gov/clone/library/genomic/426/ (BD_CBa library).

Supplementary Table 2. Correspondence between sequenced chromosomes and karyotype (in Lusinska *et al.*[1]).

B. distachyon	***B. stacei*** (assignment based on sequencing data)	***B. stacei*** (assignment based on cytogenetic criteria[1])	**Ancestral grass equivalents**
Bd1 (external part)	Bs2	Bs3	Os3
Bd1 (interstitial part)	Bs6 (35S rDNA)	Bs10 (35S rDNA)	Os7
Bd1 (central part)	Bs7	Bs2 or Bs8	Os6
Bd2 (external part)	Bs1	Bs1	Os1
Bd2 (central part)	Bs8	Bs7	Os5
Bd3 (external part)	Bs4	Bs4	Os2
Bd3 (central part)	Bs3	Bs6	Os8, Os10
Bd4 (short arm)	Bs10	Bs9	Os12, Os9, Os11
Bd4 (long arm)	Bs5	Bs5	Os12, Os9, Os11
Bd5 (35S rDNA)	Bs9	Bs2 or Bs8	Os4

Supplementary Note 1

Multiple lines of evidence (phylogenetic trees, structure analysis, k-mers) suggest that the *B. hybridum* D and S plastotypes are reproductively isolated. To experimentally test their compatibility, we conducted controlled crosses between the S plastotype line ABR113 as the female parent and the D plastotype line Bhyb26 as the male parent. We successfully created two F1 hybrids that were verified by PCR markers (Supplementary fig. 10). However, both F1 plants failed to set seed from approximately 500 flowers indicating that they are sterile. We also made the reciprocal cross, Bhyb26 as the female parent and ABR113 as the male parent, but this cross failed to produce any viable seed from 29 attempts. By contrast, crosses between S plastotype lines ABR113 and BdTR6g were successfully used to create our mapping population. Thus, infertility between D and S plastotypes likely explains the reproductive isolation of the D and S plastotype lines and the distinct genomic signatures of the D plastotype line Bhyb118-5 and the S plastotype line Bhyb118-8 that were collected at the same location.

Supplementary Note 2

Analysis of 19,805 1:1 high-confidence homeologous gene pairs with a paired t-test indicated that average log-transformed gene expression was not systematically different between the two subgenomes (p=0.40). To examine HEB on a per-gene basis, we used a likelihood ratio test implemented in DESeq2 [2], explicitly accounting for gene length and variation between libraries. In both leaves and spikes, roughly half of all testable homeolog pairs showed HEB (50% and 46%, respectively). Of the nearly 4,000 genes that were consistently biased in the same direction in all experiments, 1,938 and 1,949 genes were biased toward the S or D homeolog, respectively. Given the extreme subtlety of these differences, we cannot conclude that gene expression in *B. hybridum* is globally biased towards one subgenome. This situation is similar to that observed in a large wheat transcriptional study that did not find evidence for a single subgenome dominating overall gene expression [3]. However, they did find that single subgenomes could dominate expression for genes involved in individual traits like seed development or defense [3].

Supplementary References

1. Lusinska J, Majka J, Betekhtin A, Susek K, Wolny E, Hasterok R. Chromosome identification and reconstruction of evolutionary rearrangements in *Brachypodium distachyon, B. stacei* and *B. hybridum*. *Annals of Botany* **122**, 445-459 (2018).

2. Love MI, Huber W, Anders S. Moderated estimation of fold change and dispersion for RNA-seq data with DESeq2. *Genome Biology* **15**, (2014).

3. Ramírez-González RH*, et al.* The transcriptional landscape of polyploid wheat. *Science* **361**, eaar6089 (2018).
