# Supplementary material for: Gradual polyploid genome evolution revealed by pan-genomic analysis of Brachypodium hybridum and its diploid progenitors
Source: Nat Commun. 2020 Jul 29;11:3670. doi: 10.1038/s41467-020-17302-5 (PMC7391716; doi:10.1038/s41467-020-17302-5)
Supplement: Supplementary file 3 — Reporting Summary [file 41467_2020_17302_MOESM3_ESM.pdf]

Corresponding author(s): John Vogel

Last updated by author(s): May 6, 2020

Reporting Summary

Nature Research wishes to improve the reproducibility of the work that we publish. This form provides structure for consistency and transparency in reporting. For further information on Nature Research policies, see our Editorial Policies and the Editorial Policy Checklist.

Statistics

For all statistical analyses, confirm that the following items are present in the figure legend, table legend, main text, or Methods section.

n/a	Confirmed	
☐	☒	The exact sample size (*n*) for each experimental group/condition, given as a discrete number and unit of measurement
☐	☒	A statement on whether measurements were taken from distinct samples or whether the same sample was measured repeatedly
☐	☒	The statistical test(s) used AND whether they are one- or two-sided<br>*Only common tests should be described solely by name; describe more complex techniques in the Methods section.*
☒	☐	A description of all covariates tested
☒	☐	A description of any assumptions or corrections, such as tests of normality and adjustment for multiple comparisons
☐	☒	A full description of the statistical parameters including central tendency (e.g. means) or other basic estimates (e.g. regression coefficient) AND variation (e.g. standard deviation) or associated estimates of uncertainty (e.g. confidence intervals)
☐	☒	For null hypothesis testing, the test statistic (e.g. *F*, *t*, *r*) with confidence intervals, effect sizes, degrees of freedom and *P* value noted<br>*Give P values as exact values whenever suitable.*
☒	☐	For Bayesian analysis, information on the choice of priors and Markov chain Monte Carlo settings
☒	☐	For hierarchical and complex designs, identification of the appropriate level for tests and full reporting of outcomes
☒	☐	Estimates of effect sizes (e.g. Cohen's *d*, Pearson's *r*), indicating how they were calculated

Our web collection on statistics for biologists contains articles on many of the points above.

Software and code

Policy information about availability of computer code

Data collection	Sequence data was collected using the manufacturers software: Illumina was collected using HiSeq 2500 Control Software (HCS) v2.0.12 and Real Time Analysis (RTA) v1.18.61. PacBio sequence was collected using RSII Instrument Control Software v2.3.0 and SMRT Analysis v2.3.0
Data analysis	All software used was listed and described in the manuscript. The following programs were used: ALLPATHS (vLG); MST map (v1.0.0-2015); Meraculous assembler (2.2.2.5 release); HiRiseTM assembler; HipMer genome assembler (v0.9.6); ALLMAPS (v0.7.5); PASA (v2.3); EXONERATE (v2.4.0); RepeatMasker (v4.0.5); FGENESH+ (v2.0); FGENESH_EST (v2.6); IQTREE v. 1.6.7; Progressive Cactus (v0.1); ZEN 2.3 Pro (Zeiss); Photoshop CS3 (Adobe); NOVOPlasty v.2.7.1; blasr v.5.3.2; samtools v.1.4.1; Canu v.1.7.1; IGV v.2.3.8; MAFFT v.7.215; trimAl v.1.2rev59; Maffilter (v1.3); MafStrander (v1.0); FigTree (v. 1.4.0); STRUCTURE HARVESTER (v. 0.9.94); DISTRUCT (v. 1.1); BEAST 2.4.7 software; polyCRACKER; TRACER v.1.6; sourmash (v2.0.0); Plotly Dash (v0.30.0); GET_HOMOLOGUES-EST v09052018; Rstat package v. 4.1.0; Picard tools (v2.18); GATK (v4.0); BBtools (v. 38.0); HTSeq (v. 0.9.1); DESeq2 v. 1.24.0; bedtools (v2.27.1); Biopython (v. 1.7.0); PAML (v. 4.9h)

For manuscripts utilizing custom algorithms or software that are central to the research but not yet described in published literature, software must be made available to editors and reviewers. We strongly encourage code deposition in a community repository (e.g. GitHub). See the Nature Research guidelines for submitting code & software for further information.

Data

Policy information about availability of data

All manuscripts must include a data availability statement. This statement should provide the following information, where applicable:
- Accession codes, unique identifiers, or web links for publicly available datasets
- A list of figures that have associated raw data
- A description of any restrictions on data availability

Genome assemblies and annotations can be downloaded from Phytozome [https://phytozome.jgi.doe.gov/]. The direct link for the B. hybridum ABR113 reference

genome is [https://phytozome-next.jgi.doe.gov/info/Bhybridum_v1_1], the direct link for the B. stacei ABR114 reference genome is [https://phytozome-next.jgi.doe.gov/info/Bstacei_v1_1] and the direct link for the B. distachyon Bd21 reference genome is [https://phytozome-next.jgi.doe.gov/info/Bdistachyon_v3_1]. The other genome assemblies and annotations created in this study (listed in supplementary data 1) can be downloaded from the B. hybridum genome page [https://phytozome-next.jgi.doe.gov/info/Bhybridum_v1_1] through the download directory labelled "Additional genomes used in Gordon et al. Nat. Comm. 2020" direct link [https://genome.jgi.doe.gov/portal/pages/dynamicOrganismDownload.jsf?organism=Bhybridum]. Note that a free account is required to download data from Phytozome. The raw reads for the genomic sequences and RNA sequences are available from NCBI or ENA. The samples numbers and links are provided in Supplementary data 4 and 9. Seeds for the lines used in this study are available from the USDA National Plant Germplasm Service or by request from the authors. The source data underlying Figs 6,7 and 8 and Supplementary Figs 9,11 and 12 are provided as an Excel file named Source Data file.

Field-specific reporting

Please select the one below that is the best fit for your research. If you are not sure, read the appropriate sections before making your selection.

- [x] Life sciences
- [ ] Behavioural & social sciences
- [ ] Ecological, evolutionary & environmental sciences

For a reference copy of the document with all sections, see nature.com/documents/nr-reporting-summary-flat.pdf

Life sciences study design

All studies must disclose on these points even when the disclosure is negative.

Sample size	na
Data exclusions	no data was excluded
Replication	all replicates were included
Randomization	na
Blinding	na

Reporting for specific materials, systems and methods

We require information from authors about some types of materials, experimental systems and methods used in many studies. Here, indicate whether each material, system or method listed is relevant to your study. If you are not sure if a list item applies to your research, read the appropriate section before selecting a response.

Materials & experimental systems

n/a	Involved in the study	
x		Antibodies
x		Eukaryotic cell lines
x		Palaeontology and archaeology
x		Animals and other organisms
x		Human research participants
x		Clinical data
x		Dual use research of concern

Methods

n/a	Involved in the study	
x		ChIP-seq
x		Flow cytometry
x		MRI-based neuroimaging
