# Supplementary material for: Gradual polyploid genome evolution revealed by pan-genomic analysis of Brachypodium hybridum and its diploid progenitors
Source: Nat Commun. 2020 Jul 29;11:3670. doi: 10.1038/s41467-020-17302-5 (PMC7391716; doi:10.1038/s41467-020-17302-5)
Supplement: Supplementary file 4 — Description of Additional Supplementary Files [file 41467_2020_17302_MOESM4_ESM.pdf]

Description of Additional Supplementary Files

File name: Supplementary data 1
Description: Assembly statistics. Genome assemblies and annotations can be downloaded from Phytozome [https://phytozome.jgi.doe.gov/]. The direct link for the B. hybridum genome is [https://phytozome-next.jgi.doe.gov/info/Bhybridum_v1_1] and the direct link for the B. stacei genome is [https://phytozome-next.jgi.doe.gov/info/Bstacei_v1_1]. The other genome assemblies and annotations created in this study can be downloaded from the B. hybridum genome page [https://phytozome-next.jgi.doe.gov/info/Bhybridum_v1_1] through the download directory labelled "Additional genomes used in Gordon et al. Nat. Comm. 2020" direct link [https://genome.jgi.doe.gov/portal/pages/dynamicOrganismDownload.jsf?organism=Bhybridum] . Note that a free account is required to download data from Phytozome.

File name: Supplementary data 2
Description: *Brachypodium stacei* F2 genotypes. Genotypes of 174 F2 individuals from a mapping cross between *B. stacei* lines ABR114 and TE4.3. Yellow and blue denote homozygosity at one of the parental lines and green indicates heterozygosity. The 10 sheets correspond to the 10 genetic linkage groups and the 10 chromosomes.

File name: Supplementary data 3
Description: *Brachypodium hybridum* F2 genotypes. Genotypes of 174 F2 individuals from a amapping cross between *B. hybridum* lines ABR113 and BdTR6g. A and B indicate homozygous markers, X= heterozygous and U=unknown. The 15 sheets correspond to the 15 genetic linkage groups and the 15 chromosomes.

File name: Supplementary data 4
Description: Accession used in this study and which accessions were used in each experiment.

File name: Supplementary data 5
Description: Nexus file containing 745,845 concatenated SNVs for all lines analyzed.

File name: Supplementary data 6.
Description: Nexus file containing 5,543 concatenated SNVs for all lines analyzed.

File name: Supplementary data 7.
Description: Pan-genome matrix with gene numbers.

File name: Supplementary data 8.
Description: Pan-genome matrix with gene names.

File name: Supplementary data 9.
Description: List or RNA-seq libraries used.

File name: Supplementary data 10.
Description: Nexus file containing plastome SNPs.

File name: Supplementary data 11
Description: dN/dS ratios.
