# Supplementary material for: Gradual polyploid genome evolution revealed by pan-genomic analysis of Brachypodium hybridum and its diploid progenitors
Source: Nat Commun. 2020 Jul 29;11:3670. doi: 10.1038/s41467-020-17302-5 (PMC7391716; doi:10.1038/s41467-020-17302-5)
Supplement: Supplementary file 2 — Peer Review File [file 41467_2020_17302_MOESM2_ESM.pdf]

Editorial Note: This manuscript has been previously reviewed at another journal that is not operating a transparent peer review scheme. This document only contains reviewer comments and rebuttal letters for versions considered at *Nature Communications.*

Reviewer #2 (Remarks to the Author):

I am responding to both my previous comments (Reviewer #2) and those made by Reviewer #1. Overall, the authors did an excellent job addressing the majority of my and Reviewer #1 previous comments. The manuscript is well written for a broad audience; discussion points are all clearly described and outlined. The below point (lines 422-425) and supporting data presented by the authors is important -- largely ignored by the polyploid community. I completely agree with the authors. This paper will serve as an important reference for the polyploid community, thus I anticipate will be highly cited.

"Thus, simply comparing a polyploid genome to reference genomes from the progenitor species will greatly overestimate the changes that occurred after polyploidization and will not provide a full understanding of polyploid genome evolution."

Here are just the last two comments for you to further consider:

1. "Comparative analysis revealed that the vast majority of B. hybridum whole gene presence/absence variation was part of the standing variation in B. distachyon."

Comment: Same comments as before. If you can't establish orthology, how can you confidently conclude that the "vast majority" of PAV in Bh was part of the standing variation in Bd? I don't disagree that the data are highly supportive of your model to explain the observed subgenome differences in PAV. My recommendation would be to simply replace "revealed" with "suggest" in this line and similar statements in the manuscript.

2. "However, since polyploidization essentially duplicates all the core genes I would expect that core genes would be lost at an elevated rate relative to the diploid progenitor. That we do not observe this suggests that, in this case, there is selection to maintain intact subgenomes. This is the surprising finding."

Response: Genes are retained in duplicate following polyploidy largely to maintain proper network stoichiometry. (See review on the Gene Balance Hypothesis: https://www.pnas.org/content/109/37/14746.long). The core genome likely encodes for essential functions, thus, this finding is the predicted outcome.

3. Regarding the additional comments made by Reviewer #1: I completely agree with the authors. I am also unaware of similar studies in other polyploids, despite what was suggested by Reviewer #1. There are several novel and exciting findings presented by the author in this manuscript (which are unique from their previous work). I also agree with the authors response regarding the Tef genome paper, comment #2 and comment #3. I don't think it's necessary to cite the Tef genome paper. I will leave to the authors and editor to decide. In my opinion, the authors have adequately addressed the comments/concerns raised by reviewer #1.

Reviewer #4 (Remarks to the Author):

The authors response on most of the points I raised and the adaptations (if any) and updates in the ms. are often dissappointing and/or didn't adress the critical points I mentioned. Length and structure of the ms. remains an issue and esp. the linking of plastome type/nomenclature and genome-type is still somewhat enigmatic and not immedeatedly understandable to the non-expert.

Reviewer #5:

[Editor: This reviewer was asked to comment authors responses to previous reviewer #3's

suggestions. The detailed reviewer's comments were submitted in Remark to Editor section as summarized below.]

The reviewer thinks several of the major concerns remain unaddressed:

1) The reviewer concerned about the genome assembly approach, together with the concerns about the annotation differences among these species raised by other reviewers. The authors only stated that high quality genomes are used in this study but didn’t provide any statistics. This certainly affects the PAV and the TE/K-mer analyses.

2) The reviewer also concerned about the number and timings of origin of the diploid progenitors and the allotetraploids, which is critical to the study. This question, together with the reviewer #4’s comment about the GPS data for the collection of the individual lines, is the key to understand the true origination of the genetic diversity (SNVs) in diploid progenitors and polyploids. However, the authors didn’t answer/address the concern directly.

3) Regarding the SNP/SNVs, the author changed the term SNP to SNV, which actually still can’t distinguish the variants within the same species and the variants intraspecies.

In general, the reviewer thinks the authors can improve this manuscript by making much more solid evidences. Especially, the authors need to re-think about the divergence timing, the size and biogeography of the three populations.

Response to reviewers' comments

Our responses are highlighted green. In addition to noting significant changes below, we have uploaded files for the manuscript and methods with all changes tracked as related manuscript files.

Reviewer #2 (Remarks to the Author):

I am responding to both my previous comments (Reviewer #2) and those made by Reviewer #1. Overall, the authors did an excellent job addressing the majority of my and Reviewer #1 previous comments. The manuscript is well written for a broad audience; discussion points are all clearly described and outlined. The below point (lines 422-425) and supporting data presented by the authors is important -- largely ignored by the polyploid community. I completely agree with the authors. This paper will serve as an important reference for the polyploid community, thus I anticipate will be highly cited.

"Thus, simply comparing a polyploid genome to reference genomes from the progenitor species will greatly overestimate the changes that occurred after polyploidization and will not provide a full understanding of polyploid genome evolution."

Here are just the last two comments for you to further consider:

1. "Comparative analysis revealed that the vast majority of B. hybridum whole gene presence/absence variation was part of the standing variation in B. distachyon."

Comment: Same comments as before. If you can't establish orthology, how can you confidently conclude that the "vast majority" of PAV in Bh was part of the standing variation in Bd? I don't disagree that the data are highly supportive of your model to explain the observed subgenome differences in PAV. My recommendation would be to simply replace "revealed" with "suggest" in this line and similar statements in the manuscript.

We changed "revealed" to "suggest" as requested.

2. "However, since polyploidization essentially duplicates all the core genes I would expect that core genes would be lost at an elevated rate relative to the diploid progenitor. That we do not observe this suggests that, in this case, there is selection to maintain intact subgenomes. This is the surprising finding."

Response: Genes are retained in duplicate following polyploidy largely to maintain proper network stoichiometry. (See review on the Gene Balance Hypothesis:

https://www.pnas.org/content/109/37/14746.long). The core genome likely encodes for essential functions, thus, this finding is the predicted outcome.

The reviewer is correct that genes involved in multi-subunit complexes and some signaling pathways will be preferentially retained as duplicates after polyploidization to maintain dosage balance. The need to retain dosage balance will also promote the retention of larger genomic fragments (up to whole chromosomes) that contain these genes. However, only a fraction of all genes are constrained by dosage balance. While I suspect that that core genes are more likely to be constrained by dosage balance than shell genes, I still expect that a very large number of core genes would not be so constrained. However, I don't have firm numbers here. Thus, the expectation is that a sizeable number of core genes would be functionally redundant after polyploidization and could be lost without major consequence. This is consistent with what we know about polyploid genome evolution where the genome sheds genes (even core genes) to return to a diploid-like state with an enrichment of genes involved in complexes retaining genes from both parents. That said, this comment concerns a statement in our previous response to the reviewer and the statement in question does not appear in the manuscript so we did not make any changes. I believe we already addressed the substance of the original comment which concerned whether we could say if the PAV variants in B. hybridum were direct descendants from B. distachyon.

3. Regarding the additional comments made by Reviewer #1: I completely agree with the authors. I am also unaware of similar studies in other polyploids, despite what was suggested by Reviewer #1. There are several novel and exciting findings presented by the author in this manuscript (which are unique from their previous work). I also agree with the authors response regarding the Tef genome paper, comment #2 and comment #3. I don't think it's necessary to cite the Tef genome paper. I will leave to the authors and editor to decide. In my opinion, the authors have adequately addressed the comments/concerns raised by reviewer #1.

Reviewer #4 (Remarks to the Author):

The authors response on most of the points I raised and the adaptations (if any) and updates in the ms. are often dissappointing and/or didn't adress the critical points I mentioned. Length and structure of the ms. remains an issue and esp. the linking of plastome type/nomenclature and genome-type is still somewhat enigmatic and not immedeatedly understandable to the non-expert.

The manuscript was extensively edited for length and clarity. Major changes include:

The manuscript was shortened from 7,013 to 5,821 words (introduction, results, discussion) to conform to the limit of 6,500 set by the editor.

The number of references was reduced from 109 to 58.

A new figure (now figure 1) was added to graphically explain the plastome/genome nomenclature.

The plastome structure portion was removed, including all text and figure 4b, because it was not essential to our overall conclusions and the reviewer's objections.

The Online Methods and Results were placed in a different file for clarity and to remove the methods' references from the main text.

Reviewer #5:

[Editor: This reviewer was asked to comment authors responses to previous reviewer #3's suggestions. The detailed reviewer's comments were submitted in Remark to Editor section as summarized below.]

The reviewer thinks several of the major concerns remain unaddressed:

1) The reviewer concerned about the genome assembly approach, together with the concerns about the annotation differences among these species raised by other reviewers. The authors only stated that high quality genomes are used in this study but didn't provide any statistics. This certainly affects the PAV and the TE/K-mer analyses.

We added the following text to the methods to address concerns about assembly quality:

Regarding the k-mer analysis on page 17-18

"While variation in the completeness of the genome assemblies can impact this analysis, we do not believe this affected our main conclusions for three reasons. First, 13 of the 17 lines analyzed were similar quality Illumina assemblies. Second, the higher quality assemblies always grouped together consistently with the most closely related lines. For example, all six *B. hybridum* S plastotype lines show very similar patterns despite the fact that Bd28 was a PacBio assembly, ABR113 was a high-quality reference assembly and the other four lines were Illumina assemblies. Third, the subgenomes in each *B. hybridum* line had very different k-mer patterns. That said, the relative abundance of k-mers in classes six and seven may be affected by assembly quality since the lines with the highest abundance of those k-mers, Bd21, Bd28, ABR113 are also the lines with the best assemblies. "

Regarding the pan-genome analysis on page 20

"While assembly quality could affect the pan-genome analysis, we do not think this caused significant bias because low complexity sequences like genes are efficiently assembled in the compact *Brachypodium* genomes. In addition, the fact that the three higher quality assemblies appear

indistinguishable from the other assemblies indicates that assembly quality is not a systematic source of error."

We added the following information to Supplementary table 1 to provide more information about the assembly quality:

total assembly % of reference. Note that the "total assembly % of reference" ranges from 99-104% indicating that all the assemblies contain nearly the same amount of sequence as the corresponding reference genomes.

contig N50/L50

contig N90/L90

percent GC

2) The reviewer also concerned about the number and timings of origin of the diploid progenitors and the allotetraploids, which is critical to the study. This question, together with the reviewer #4's comment about the GPS data for the collection of the individual lines, is the key to understand the true origination of the genetic diversity (SNVs) in diploid progenitors and polyploids. However, the authors didn't answer/address the concern directly.

In our last revision, we included the GPS coordinates for all lines in Supplementary table 2. To further address the reviewer's concerns, we have added a figure (Supplementary figure 4) graphically showing the collection locations of all lines used in the study. From this figure, you can see that the number of B. hybridum and B. stacei lines is very low and they come from a smaller geographic area than the B. distachyon lines. Furthermore, in previous publications we showed that the B. distachyon group (EDF clade) that is most closely related to the S plastotype B. hybridum lines is distributed across the entire range of B. distachyon. We like emphasize that our analysis is not meant to be a population analysis of B. hybridum diversity because we only have a handful of collection B. hybridum collection sites and usually only one individual from each site. On pages 12-13 we removed a paragraph about the placement of the new B. distachyon lines into various clades because it wasn't central to our main conclusions about polyploidy and it may have been confusing this matter.

Regarding the "number and timings of origin of the diploid progenitors and the allotetraploids" We are not sure what more the reviewer wants to see. Our previous published work has already thoroughly established the presence and nature of the major sub-groups of B. distachyon. We conclusively placed the B. hybridum D subgenomes into that context using an enormous SNP set. We dated the divergence times using the most advanced and current methods. If there are specific concerns please bring them to our attention, otherwise, we are at a loss as to what more the reviewer wants.

3) Regarding the SNP/SNVs, the author changed the term SNP to SNV, which actually still can't distinguish the variants within the same species and the variants intraspecies.

To further clarify this, we changed the text of the first paragraph of "**Nuclear phylogenomics, genome structure and SNV-based divergence dating analysis"** results section to include the exact number of base pairs that are polymorphic in B. distachyon and B. stacei.

The paragraph now reads as follows:

"Phylogenomic analysis was conducted with eight *B. hybridum* lines (16 subgenomes), 99 *B. distachyon* lines and one *B. stacei* line (Supplementary table 2, Supplementary fig. 4) using 745,858 orthologous nucleotide positions in all lines. These were selected because they were all polymorphic (SNVs) between at least one pair of *B. distachyon* lines. Fifteen percent (114,694 of 745,858) of these were polymorphic (SNVs) between at least one pair of *B. stacei*/S subgenomes (Supplementary file 1). The best maximum likelihood (ML) IQTREE tree contained a main split between *B. stacei* and *B. distachyon* and the root placement of this split was confirmed using a subset of 5,443 nucleotide positions for which we could identify corresponding orthologous loci in *Oryza sativa* (Fig. 4a, Supplementary figs. 5, 6, Supplementary file 2). Fourteen percent of these (779 of 5,443) were polymorphic (SNVs) between at least one pair of *B. stacei*/S subgenomes."

In addition, we added supplementary files (supplementary file 1 and 2) that contain the concatenated nucleotides for both SNV sets so anyone can see the exact nucleotides in each line.

In general, the reviewer thinks the authors can improve this manuscript by making much more solid evidences. Especially, the authors need to re-think about the divergence timing, the size and biogeography of the three populations.

Please see our response to 2 above. To reiterate, divergence timing was done by current best practices and the population structure for B. distachyon is well established and already published (Gordon et al. 2017). In any case, our analysis clearly indicate that there were 2 events that formed B. hybridum, one even happened relatively recently, one happened about a million years ago, and that the individuals we examined have been genetically isolated for a long time.

Reviewers' comments:

Reviewer #2 (Remarks to the Author):

The authors successfully addressed my primary concerns and comments. This paper will serve not only as an important paper for the polyploid community, but also for the broader genomics community as the focus has been shifting towards the importance of analyzing pangenomes.

Reviewer #4 (Remarks to the Author):

Gordon et al., Gradual polyploid genome evolution ....

Thanks for the response on my previous comments and thanks for the exhaustive work on restructuring the ms and rewriting large portions of the ms. I really appreciate this. I reread through the ms.. It now reads much better than the initial versions, the introduction is much more focussed and the discussion got a very impressing overhaul. Nevertheless I still have some points I would like to raise:
line 116: not all of our crops are outbreeders as suggested in this sentence. Think of cereals
line 138: what are comparative grde genome assemblies?. Unclear
line 145: post-polyploidisation gene loss is still in its infancy (after 1.4 My). Hmm, some cereal studies suggest that this is a very rapid process. Only few generations. Is the basic hypothesis wrong? Does out- vs. inbreeding behaviour have impact? Btw: any knowledge about subgenome recombination in Brachypodium hybridum? This might have impact on some of the aspects you analyse later on...
line 180: what are comparative assemblies?
line 320: referenvce to suppl figure 9a. Should be 9b (?). However in the figure legend it gives 8b... Please fix
line 332: refe to suppl figure 9b. Should be 9c (8c?)
line 341: Has a reverese cross been done? So swapping of female and male parent.
line 366 ff: I'm not sure I get the message here. Can you simplify?
line 372 ff: Selection analysis. The thresholds used are sort of non-traditional thresholds. More important the conclusion basically is that no evidence for selection has been found. The whole section could be removed or moved into supplement.
line 424 ff: "extremly subtle bis" is an interesting wording. Basically there is no difference. Please shorten this section to aminimum
line 503: 7 to 2-1 Ma... ???? There is prob atypo but I cannot really decipher what this is supposed to tell me

Figures: In general The quality of the figures is disputable and not always illustrates the point made in an appropriate manner:
why is class 3 missing from figure 6?
figure 7 asks for some art work. So does fig 4 a and fig 5. There is tendency for info overload in the figures... (I'm well aware about the huge amount of data that was used for this work. Nevertheless, sometimes a story is better be told by less info-dense figures.

Again thanks and congratulations for the insightful work!

Reviewer #5 (Remarks to the Author):

This is the revised version of a manuscript that I have reviewed a while ago. After a careful reading of the new version, I acknowledge that the authors did a good job of revision, and have addressed all of the major concerns raised by previous reviewer #3. I have no additional questions.

Yuannian Jiao

Response to reviewer's comments
Author responses in green.

Reviewer #4 (Remarks to the Author):

Gordon et al., Gradual polyploid genome evolution ....

Thanks for the response on my previous comments and thanks for the exhaustive work on restructuring the ms and rewriting large portions of the ms. I really appreciate this. I reread through the ms.. It now reads much better than the initial versions, the introduction is much more focussed and the discussion got a very impressing overhaul. Nevertheless I still have some points I would like to raise:
line 116: not all of our crops are outbreeders as suggested in this sentence. Think of cereals

We appreciate the comment of the reviewer. The sentence contained "and/or" to indicate that not all of the qualities listed applied to all polyploidy crops. To further clarify, the sentence has been rewritten as follows "Unfortunately, polyploid biomass and grain crops are difficult experimental subjects because of their large, complex genomes; large physical size; and, for biomass crops, their typically outbreeding nature [24].

line 138: what are comparative grde genome assemblies?. Unclear

We changed the text to read "lower quality assemblies".

line 145: post-polyploidisation gene loss is still in its infancy (after 1.4 My). Hmm, some cereal studies suggest that this is a very rapid process. Only few generations. Is the basic hypothesis wrong? Does out- vs. inbreeding behaviour have impact?

Evolution is clearly happening much more slowly than we expected in the Brachypodium species and lines we have studied so far. Whether this is due to the biology of the system or the fact that previous studies did not use a pan-genomic approach will have to be answered by future studies in other systems.

Btw: any knowledge about subgenome recombination in Brachypodium hybridum? This might have impact on some of the aspects you analyse later on...

There is no evidence to support extensive recombination between subgenomes of the reference line, ABR113, as evidenced by our synteny and k-mer analyses. This is not surprising since the diploid progenitors diverged over 10 million years ago and they have different chromosome numbers and divergent sequence. That said, we cannot rule out the swapping of very small segments between subgenomes at this time.

line 180: what are comparative assemblies?

We changed this to lower-quality assemblies.

line 320: referenvce to suppl figure 9a. Should be 9b (?). However in the figure legend it gives 8b... Please fix

Fixed. Thanks for catching that.

line 332: refe to suppl figure 9b. Should be 9c (8c?)

Fixed. Thanks for catching that.

line 341: Has a reverese cross been done? So swapping of female and male parent.

Yes we conducted the reciprocal cross. We added the following sentence to the manuscript to indicate this:
"We also made the reciprocal cross, Bhyb26 as the female parent and ABR113 as the male parent, but this cross failed to produce any viable seed from 29 attempts."

line 366 ff: I'm not sure I get the message here. Can you simplify?

The text was changed to:

"To compare this to the intraspecific variation in *B. distachyon*, we determined how many pan-genes in each *B. distachyon* line were missing from only that line. Since these genes would have been core genes if the pan-genome was constructed without that particular line, this is the appropriate comparison to the number of *B. distachyon* core genes missing from the *B. hybridum* D subgenomes. Since the number of genes missing from only one *B. distachyon* line was similar to the number of core genes missing from the *B. hybridum* D subgenomes, the core genes missing from the *B. hybridum* D subgenomes as a whole could simply have been missing from the actual genomes of the *B. distachyon* parents of the polyploids (Fig. 7)."

From:
"However, looking at the number of genes per *B. distachyon* line that were missing from only one *B. distachyon* line (these genes would have been core genes if the pan-genome was constructed without that particular line) revealed that the number of core genes missing from the *B. hybridum* D subgenomes as a whole could simply have been missing from the actual genomes of the *B. distachyon* progenitor species of the polyploids (Fig. 7)."

line 372 ff: Selection analysis. The thresholds used are sort of non-traditional thresholds.

More important the conclusion basically is that no evidence for selection has been found. The whole section could be removed or moved into supplement.

Yes the conclusion is that there is no selection. This is a surprising finding that is important because it is another measure that the genomes are evolving slowly. We shortened this section and removed mention of our non-traditional thresholds.

line 424 ff: "extremly subtle bis" is an interesting wording. Basically there is no difference. Please shorten this section to aminimum

This was removed.

line 503: 7 to 2-1 Ma... ???? There is prob atypo but I cannot really decipher what this is supposed to tell me

We deleted ", 7 to 2-1 Ma"

Figures: In general The quality of the figures is disputable and not always illustrates the point made in an appropriate manner:

why is class 3 missing from figure 6?

Classes 3 and 7 were placed in supplementary figure 9 so that the graphs for the other classes could be made larger and easier to read. Since they are similar to other classes, they are not needed to illustrate the biological trends discussed in the paper.

figure 7 asks for some art work. So does fig 4 a and fig 5. There is tendency for info overload in the figures... (I'm well aware about the huge amount of data that was used for this work. Nevertheless, sometimes a story is better be told by less info-dense figures.

We agree that Figure 4a is detailed, however, the granularity is necessary to show the evidence supporting the major differences between the different plastotypes of B. hybridum based on their nuclear subgenomes. We have already condensed the portion of the tree containing most of the *B. distachyon* lines. Since figure 4c is essentially a cartoon version of 4a, we prefer to keep this figure unchanged.

The inset in figure 5 is artwork to summarize the overall relationship between plastomes but it is too schematic; this is why we have represented in the main figure a larger but also summarized tree showing the branch lengths within the D-type plastomes clade (e.g., showing the earlier divergence of the B. hybridum D-plastotype plastomes with respect to the B. distachyon plastomes) and the chloroplast capture events detected among the three main B. distachyon groups. We think it clearly illustrates the results in a very direct and simple way.

Figure 7 consists of simple bar plots. It includes all the samples studied in the pangenome analysis and the results could be seen at a first sight. We do not know how to convey the information in a simpler manner. We could collapse some of the *B. distachyon* lines in the middle of the graphs, as we did in figure 8, but we really don't think this would improve the figure.

Again thanks and congratulations for the insightful work!

We thank the reviewer for helping improve the manuscript.

Reviewer #4 (Remarks to the Author):

Thanks for the repeated round of revisions. All of my points raised and concerns raised have been sufficiently adressed. Thanks for interesting study and story!

Klaus Mayer
